# A Pipeline for Reconstructing Somatic Copy Number Alternation’s Subclonal Population-Based Next-Generation Sequencing Data

**DOI:** 10.3389/fgene.2019.01374

**Published:** 2020-02-27

**Authors:** Yanshuo Chu, Chenxi Nie, Yadong Wang

**Affiliations:** Center of Bioinfomatics, School of Computer Science and Technology, Harbin Institute of Technology, Harbin, China

**Keywords:** somatic copy number alternation, subclonal reconstruction, subclonal frequency, absolute copy number, bias correction

## Abstract

State-of-the-art next-generation sequencing (NGS)-based subclonal reconstruction methods perform poorly on somatic copy number alternations (SCNAs), due to not only it needs to simultaneously estimate the subclonal population frequency and the absolute copy number for each SCNA, but also there exist complex bias and noise in the tumor and its paired normal sequencing data. Both existing NGS-based SCNA detection methods and SCNA’s subclonal population frequency inferring tools use the read count on radio (RCR) of tumor to its paired normal as the key feature of tumor sequencing data; however, the sequencing error and bias have great impact on RCR, which leads to a large number of redundant SCNA segments that make the subsequent process of SCNA’s subclonal population frequency inferring and subclonal reconstruction time-consuming and inaccurate. We perform a mathematical analysis of the solution number of SCNA’s subclonal frequency, and we propose a computational algorithm to reduce the impact of false breakpoints based on it. We construct a new probability model that incorporates the RCR bias correction algorithm, and by stringing it with the false breakpoint filtering algorithm, we construct a whole SCNA’s subclonal population reconstruction pipeline. The experimental result shows that our pipeline outperforms the existing subclonal reconstruction programs both on simulated data and TCGA data. Source code is publicly available as a Python package at https://github.com/dustincys/msphy-SCNAClonal.

## Introduction

Tumor heterogeneity introduces challenges in cancer tissue diagnosis and subsequent treatment (Nowell, 1976). Tumor heterogeneity cannot be inferred by the properties of biomolecular through the ontology or pathway analysis (Cheng et al., 2017; Cheng et al., 2018c), but could be inferred by measuring the quantity of biomoleculars (Cheng et al., 2018b; Cheng et al., 2018d; Cheng et al., 2019). To decipher cell composition in bulk cells, somatic copy number alternations (SCNAs), most commonly found in tumor cells (Beroukhim et al., 2010), are utilized as the representative to determine tumor subclonal populations in a tumor–normal tissue paired manner (Oesper et al., 2013; Li and Xie, 2015). The benefit of using SCNA to conduct subclonal reconstruction is that the WGS data doesn’t have to be deeply sequenced (Li and Xie, 2015), because SCNA affects large, multi-kilobase-sized or megabase-sized regions of the genome, which allows the average copy number of these regions to be accurately estimated with whole genome sequencing (WGS) (Deshwar et al., 2015).

SCNA’s subclonal reconstruction algorithms attempt to infer the population structure of heterozygous tumors based on the subclonal population frequency of SCNA (Deshwar et al., 2015). However, the cellular prevalence and the absolute copy number are intertwined and next-generation sequencing (NGS)-based subclonal reconstruction needs to simultaneously estimate population frequency and the absolute copy number for each SCNA. The solution space of subclonal frequency of SCNA remains poorly understood, and there might exist multiple solutions for subclonal frequency for some SCNAs (Oesper et al., 2013), which makes the infinite site assumptions (ISAs) (Kimura, 1969; Hudson, 1983; Jiao et al., 2014) invalid. ISA is the commonly accepted and powerful assumption, which posits that each mutation occurs only once in the evolutionary history of the tumor.

To infer the SCNA’s subclonal population frequency based on NGS data, the location of SCNAs in the genome needs to be obtained first. The SCNA breakpoints are detected through multiple bin-merging processes, during which rcr of tumor to its paired normal is used as a key feature (Xi et al., 2010). However, the sequencing error and bias have great impact on RCR, which leads to false positive breakpoints and incorrect subclonal reconstruction (Please refer to **Figures S2** and **S3**, **Tables S2** and **S3** in the **Supplementary**). The higher sensitivity the SCNA detection tools show, the more prone to the sequencing error the tools would be. For example, BIC-seq (Xi et al., 2010) first splits whole genome into small bins, then uses the Bayesian Information Criterion as the bin merging and stopping criterion to detect SCNA breakpoints. When sensitivity parameter λ of BIC-seq is very high, the true positive rate and the false discovery rate will decrease simultaneously (Xi et al., 2010), which means the SCNA regions will be separated into small fragments by the false positive breakpoints (Xi et al., 2010). The choice of parameter λ is equivalent to setting type I error; in other words, when performing the loop of combining windows, two neighboring windows that should be combined are left separated apart. Since the reconstruction algorithm of subclone depends on the proportion of subclone populations of somatic mutation to define mutation set and its subpopulation (Deshwar et al., 2015) (Please refer to **Figure S4** for the definition of subpopulation and subclonal population), in order to more precisely estimate the subclonal population ratio of every SCNA fragment, we need to choose a smaller λ to ensure the high true positive rate of breakpoints, so as to more accurately estimate the subclonal population frequency. However, the false positive breakpoints split the SCNA regions into many small SCNA fragments, which violates ISA and results in many redundant input data and causes the subclone reconstruction process to be extremely slow and time consuming.

Existing (NGS) based subclonal reconstruction methods, such as ThetA (Oesper et al., 2013) and Mixclone (Li and Xie, 2015), use expectation maximation (EM) or maximum likelihood method (MLM) to infer the subclonal frequency and the absolute copy number of every input data. To reduce the searching space, MixClone assumes that the number of subclonal population is less than 3, and this number (1 or 2) needs to be predefined. During the maximization step of the EM process, MixClone assumes the subclonal frequencies of all the subclonal population only equal to several combinations of discrete values to further reduce the searching space. Thus, MixClone’s accuracy is compromised for speed of computation. On the other side, Theta (Oesper et al., 2013) does not make any compromise on searching space. Thus, Theta is extremely time consuming while search optimal subclonal frequency in (0,1) for every input data, which makes it unable to perform subclonal reconstruction for more than three subclonal populations.

With the ever increasing data of biotechnology comes the chance of developing computational toolkit (Cheng et al., 2016; Cheng et al., 2018a; Cheng et al., 2019) to find out the pathogeny of diseases; in this article, we provide a pipeline for reconstructing SCNA’s subclonal population-based NGS data. We first perform a mathematical analysis of the solution number of SCNA’s subclonal frequency, propose and prove the theorem of solution number of SCNA’s subclonal frequency, and present a method to filter out false SCNA breakpoints based on it. Then we propose a probability model that incorporates rcr bias correction algorithm we previously developed, and we construct an SCNA’s subclonal population reconstruction pipeline by stringing it with the false breakpoint filtering algorithm. We model the read depth of tumor sample as a Poisson distribution with the expected tumor read count proportional to the absolute copy number and subclonal frequency. We use the tree-structured stick breaking Dirichlet process (Prescott Adams et al., 2010) to generate the tree structure of tumor’s evolutionary history, and use the Markov Chain Monte Carlo (MCMC) to obtain the result of subclonal reconstruction. The experimental result shows that our pipeline outperforms the existing subclonal reconstruction programs both on simulated data and TCGA data.

## Materials and Methods

### Solution Space of SCNA’s Subclonal Population Frequency

The RCR and the b-allele frequency (BAF) of the heterozygous single nucleotide polymorphism (SNP) locus in the SCNA segment are commonly used as input for the sequencing data-based SCNA’s copy number and subclonal frequency inferring tools (Wang et al., 2007; Oesper et al., 2013; Li and Xie, 2015). Since the number of reads mapped in certain genome region is proportional to the copy number of this region, the RCR is set to be proportional to $\frac{{\bar{C}}_{j}}{2}$ by existing tools (Oesper et al., 2013; Li and Xie, 2015), where $\frac{{\bar{C}}_{j}}{2}$ denotes its average copy number of the *j*th SCNA segment. Let *ϕ_j_* denote the subclonal population cellular prevalence of the *j*th SCNA segment; $C_{j}^{\text{T}}$ denote its absolute copy number; $\mu_{jk}^{\text{T}}$ represent the BAF of the *k*th heterozygous SNP locus in the *j*th SCNA segment; ${\bar{\mu }}_{j}$ represent the average BAF of the *k*th heterozygous SNP locus in the *j*th SCNA segment. Then we have the following equation set

(1) $$\{\begin{array}{l} {\bar{C}}_{j}=\phi_{j}*C_{j}^{T}+(1-\phi_{j})*2,\\ {\bar{C}}_{j}=\frac{1}{{\bar{\mu }}_{jk}}\left[\phi_{j}*C_{j}^{T}*\mu_{jk}^{T}+(1-\phi_{j})*2*\frac{1}{2}\right],\;k=1,\ldots ,K_{j}. \end{array}$$

where *K_j_* is the total number of heterozygous SNP loci in the *j*th SCNA segment. Since the B allele locates either in paternal or maternal haploid, both $\mu_{jk}^{\text{T}}$ and $\left(1\;-\mu_{jk}^{\text{T}}\right)$ could possibly be the BAF value in the same SCNA fragment and both ${\bar{\mu }}_{jk}$ and $\left(1-{\bar{\mu }}_{jk}\right)$ could possibly be the average BAF value in the same SCNA fragment. To reduce the complexity, we use ${\hat{\mu }}_{jk}^{\text{T}}$ to denote the smaller one of $\mu_{jk}^{\text{T}}$ and $\left(1\;-\mu_{jk}^{\text{T}}\right)$; ${\hat{\bar{\mu }}}_{jk}$ to denote the smaller one of $\mu_{jk}^{\text{T}}$ and $\left(1-{\bar{\mu }}_{jk}\right)$. Here we give a theorem to help answer the solution space of equation set 1 and we prove it in the **Supporting Information**.

Theorem 1. *Given* ${\bar{C}}_{j}$ *and* ${\left\{{\hat{\bar{\mu }}}_{jk}\right\}}_{k=1}^{K_{j}}$ *and let* $\xi =\frac{C_{j}^{\text{T}}{\hat{\mu }}_{jk}^{\text{T}}-1}{C_{j}^{\text{T}}-2}$ *, we have the following conclusions:*

1. *If* ${\bar{C}}_{j}$ *< 2, there is only one solution ϕ_j_ in Equation set 1.*
2. *If* ${\bar{C}}_{j}$ *> 2 and* ${\bar{C}}_{j}<\frac{1}{{\hat{\bar{\mu }}}_{jk}}$ *there is only one solution of ϕ_j_ in Equation set 1.*
3. *If* ${\bar{C}}_{j}$ *> 2 and* ${\bar{C}}_{j}\geq \frac{1}{{\hat{\bar{\mu }}}_{jk}}$ *, there are infinite solutions of ϕ_j_ in Equation set 1.*
4. *If* ${\bar{C}}_{j}$ *> 2 and* ${\bar{C}}_{j}\geq \frac{1}{{\hat{\bar{\mu }}}_{jk}}$ *, there are multiple solutions of ϕ_j_ in Equation set 1 on the curves of the family of function* ${\hat{\bar{\mu }}}_{jk}=\xi \left(1-\frac{2}{{\bar{C}}_{j}}\right)+\frac{1}{{\bar{C}}_{j}}$ *, under the restriction of maximum absolute copy number C_max_. Suppose segment s_j__′_ and s_j__″_ are the two solutions for given* ${\bar{C}}_{j}$ *and* ${\left\{{\hat{\bar{\mu }}}_{jk}\right\}}_{k=1}^{K_{j}}$ *, then* $\frac{C_{j^{\prime }}^{\text{T}}{\hat{\mu }}_{j^{\prime }k}^{\text{T}}-1}{C_{j^{\prime }}^{\text{T}}-2}=\frac{C_{j^{\prime \prime }}^{\text{T}}{\hat{\mu }}_{j^{\prime \prime }k}^{\text{T}}-1}{C_{j^{\prime \prime }}^{\text{T}}-2}$ *. The multiple solution area would be* ${\bar{C}}_{j}$ ∈ (2, min(*C_j′_*, *C_j″__)_*) and ${\hat{\bar{\mu }}}_{jk}\in (\min({\hat{\mu }}_{j^{\prime }k}^{\text{T}},{\hat{\mu }}_{j^{\prime \prime }k}^{\text{T}}),2)$.

**Figure 1:**
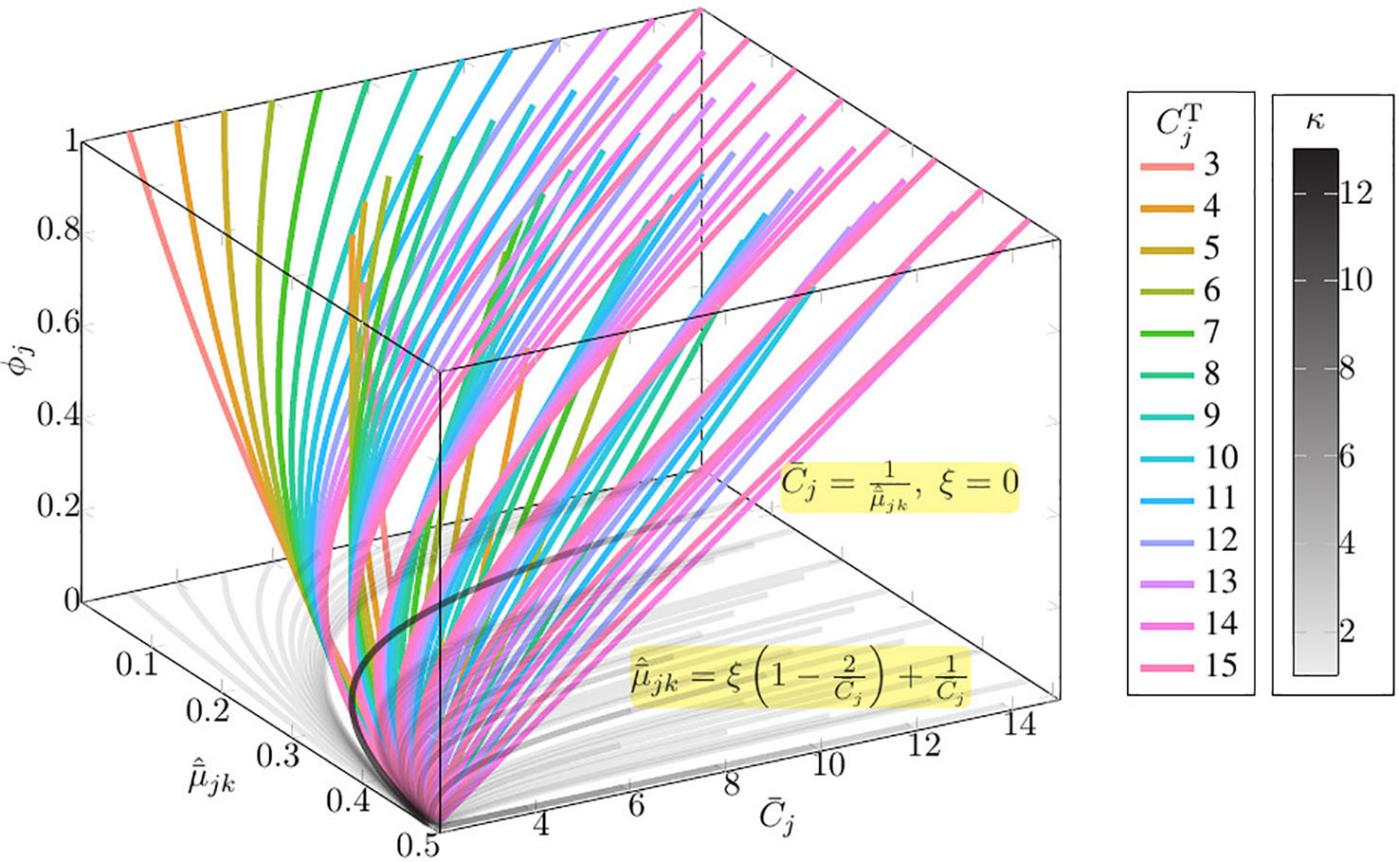
The solution space of Equation set 1 given the observation value ${\bar{C}}_{j}$ and ${\hat{\bar{\mu }}}_{jk}$ and maximum copy number *C*_max_ = 15. In this figure, κ denotes the number of solutions; $\xi =\frac{C_{j}^{T}{\hat{\mu }}_{jk}^{T}-1}{C_{j}^{T}-2}$, where $C_{j}^{T}$ is the absolute copy number of SCNA in the *j*th segment *s*_*j*_, ${\hat{\mu }}_{jk}^{\text{T}}$ is the normalized BAF of tumor reads mapped at the *k*th heterozygous SNP loci in the *j*th segments *s*_*j*_; ${\hat{\bar{\mu }}}_{jk}$ denotes the normalized average tumor reads mapped at the *k*th heterozygous SNP loci in the *j*th segments s*_j_*; ${\bar{C}}_{j}$ denotes the average copy number of segment s*_j_*; *ϕ_j_* denotes the subclonal frequency of segment s*_j_*.

As shown in **Figure 1**, given the observation value ${\bar{C}}_{j}$ and ${\hat{\bar{\mu }}}_{jk}$ and maximum copy number *C*_max_ = 15, only 7/43 of the curves of the family of function ${\hat{\bar{\mu }}}_{jk}=\xi \left(1-\frac{2}{{\bar{C}}_{j}}\right)+\frac{1}{{\bar{C}}_{j}}$ present multiple *ϕ_j_* solutions (Please refer to **Table S1** for the detail information of multi-solution range).

### The Algorithm of Filtering Out False Positive SCNA Breakpoints

We assume that there are no two adjacent SCNAs that present the same ${\bar{C}}_{j}$ and ${\hat{\bar{\mu }}}_{jk}$ and meanwhile the different *ϕ_j_* and $C_{j}^{\text{T}}$ according to Theorem 1. We use the same method described in Li and Xie (2015) to model the read count ratio of tumor and its paired normal. Based on the Lander–Waterman model (Lander and Waterman, 1988), the probability of sampling a read from a given segment depends on three main factors: 1) its copy number, 2) its total genomic length, and 3) its mappability, which depends on factors such as repetitive sequence and GC content (Li and Xie, 2015). For each segment *j*, we associate a coefficient *j*) to account for the effect of its mappability and genomic length. Thus, the expected tumor read counts mapped to segment *j*, which is denoted as λ*_j_*, are proportional to ${\bar{C}}_{j}\theta_{j}$. For example, for segment *x* and segment *y*, we have

(2) $$\frac{\lambda_{x}}{\lambda_{y}}=\frac{{\bar{C}}_{x}\theta_{x}}{{\bar{C}}_{y}\theta_{y}}$$

Because the mappability coefficients matter only in a relative sense, we take $\theta_{x}/\theta_{y}=D_{x}^{\text{N}}/D_{y}^{\text{N}}$, as these segments should have the same sequence properties between the normal and tumor samples. Thus, Equation 2 is transformed into

(3) $$\log(\lambda_{x}/D_{x}^{\text{N}})-\log(\lambda_{y}/D_{y}^{\text{N}})=\frac{{\bar{C}}_{x}}{{\bar{C}}_{y}}.$$

However, our previous study (Chu et al., 2017a) has shown the RCR of tumor to its paired normal presents a log-linear GC content bias, and has described a bias correction software “Pre-SCNAClonal” (Chu et al., 2017a) to correct this bias specifically. Let $\hat{D_{i}^{\text{S}}/D_{i}^{\text{N}}}$denote the corrected read count ratio of tumor sample and its paired normal, and let Φ() denote the bias correction process. Then we have $\hat{D_{i}^{\text{S}}/D_{i}^{\text{N}}}=\text{Φ}\left(D_{i}^{\text{S}}/D_{i}^{\text{N}}\right)$ and

(4) $$\log\left(\hat{D_{i}^{\text{S}}/D_{i}^{\text{N}}}\right)-\log\left(\hat{D_{j}^{\text{S}}/D_{j}^{\text{N}}}\right)=\log\frac{{\bar{C}}_{i}}{{\bar{C}}_{j}}.$$

Then we use the following steps to filter out false positive SCNA breakpoints.

1. First, BIC-Seq with a small λ is used to detect SCNA breakpoints. Then the whole genome is separated into SCNA fragments by these breakpoints. We use ${\left\{s_{j}\right\}}_{j=1}^{J}$ to denote this SCNA fragment set.
2. Next, Pre-SCNAClonal (Chu et al., 2017a) is used to correct the bias of RCR.
3. Next, the hierarchical clustering algorithm is used to cluster ${\left\{s_{j}\right\}}_{j=1}^{J}$ based on $\log\;\left(\hat{D_{j}^{\text{S}}/D_{j}^{\text{N}}}\right)$ of every segment with the maximum amount of cluster predefined as *C_max_* * τ, where τ is the number of subclonal populations. Suppose in this step, there are *N* clusters obtained by the hierarchical clustering algorithm. We denote the *n*th cluster as $S_{n}$ where *n* = 1, 2,…, *N*. For convenience, we call this step the aggregation step.
4. Next, the MeanShift algorithm is used to perform an unsupervised cluster search on $\cup_{s_{j}\in S_{n}}{\left\{{\hat{\bar{\mu }}}_{jk}\right\}}_{k=1}^{K_{j}}$, where $S_{n}$ is obtained by step 3. Assume there are *M_n_* BAF clusters detected in $\cup_{s_{j}\in S_{n}}{\left\{{\hat{\bar{\mu }}}_{jk}\right\}}_{k=1}^{K_{j}}$, and we use $\Psi ({\hat{\bar{\mu }}}_{jk})\in \{1,\ldots ,M_{n}\}$ to represent the cluster index. Then for every *s*_*j*_ ∈ $S_{n}$ we define the BAF cluster of *s* *_j_* to be the BAF cluster of ${\left\{{\hat{\bar{\mu }}}_{jk}\right\}}_{k=1}^{K_{j}}$ with the largest number. Then each $S_{n}$ is split into subclusters ${\left\{S_{n,m}\right\}}_{m=1}^{M_{n}}$ based on the BAF cluster of each *s*_*j*_. For convenience, we call this step the decomposition step.
5. For each $S_{n,m}$, *n* = 1,2,…,*N*, *m* = 1,2,..,*M*_*n*_, we merge two adjacent SCNA fragments, which are on the same chromosome and the distance between them is less than a predefined threshold *ρ*.

The space complexity of the algorithm of filtering out false positive SCNA breakpoints is *o*(*J* ^2^). The computational complexity of “MeanShift” and “hierarchical clustering” are $o(\sum_{n=1}^{N}{\left(I_{n}*\sum s_{j}\in S_{n}K_{j}\right)}^{2})$ and *o*(*J* ^3^), where *I* *_n_* is the number of iterations for $S_{n}$. Thus. the time complexity of the algorithm of filtering out false positive SCNA breakpoints is $o(J^{3}+\sum_{n=1}^{N}{\left(I_{n}*\sum_{s_{j}\in S_{n}}K_{j}\right)}^{2})$. The detail validation of this algorithm are described in Section 4 in the **Supplementary** (Please refer to **Figures S5–S8** for the results).

### Normal Segments Detection Method

The task of normal segments detection is to find out all the segments that ${\bar{C}}_{j}=2$, since the copy number $C_{j}^{\text{N}}$ in *s_j_* in normal sample equals 2, normally. A cancer genome differs from the reference genome by gains and losses of segments, or intervals, of the reference genome (Oesper et al., 2013).

However, due to two different sequencing processes and the coverage may not exactly be the same for tumor and its paired normal, $\hat{D_{j}^{\text{S}}/D_{j}^{\text{N}}}$ does not always equal to 1 for the normal segments (Li and Xie, 2015). In this paper, we use the same normal segments detection method described in our previous work (Chu et al., 2017a), which utilizes BAF information to detect normal segments.

Equation set 1 implies following conclusion

(5) $$\begin{array}{l} \phi_{j}=0\text{ or }C_{j}^{\text{T}}=2\Leftrightarrow {\bar{C}}_{j}=2,\\ \phi_{j}=0\text{ or }C_{j}^{\text{T}}=0\text{ or }\mu_{jk}^{\text{T}}=\frac{1}{2}\Leftrightarrow {\bar{\mu }}_{jk}^{\text{T}}=\frac{1}{2}. \end{array}$$

We detect the normal segments ${\mathbb{N}}_{t_{m}}$ from $S_{t_{m}}$ according to Equation 5 by the following two steps. First, we filter out all the segments ${\text{s}}_{j}\in S_{t_{m}}$ with ${\bar{\mu }}_{jk}^{\text{T}}\neq \frac{1}{2}$ for $k=1,\ldots ,K_{s_{j}}$. In the remaining segments, the possible $C_{j}^{\text{T}}$ could be any one in {0, 2, 4,…}, since all the possible genotypes $G_{jk}^{\text{T}}$ of allele at the *k*th site for $\mu_{jk}^{\text{T}}=\frac{1}{2}$ could be any one in {∅, PM, PPMM,…}. Next, we obtain all the normal segments ${\mathbb{N}}_{t_{m}}$ from these segments by selecting the segments with the read depth $d_{jk}^{\text{S}}$ at the *k*th heterozygous SNP site equal to the coverage of the aligned WGS data of the tumor sample.

### The Probability Model of Subclonal Population Frequency

**Figure 2:**
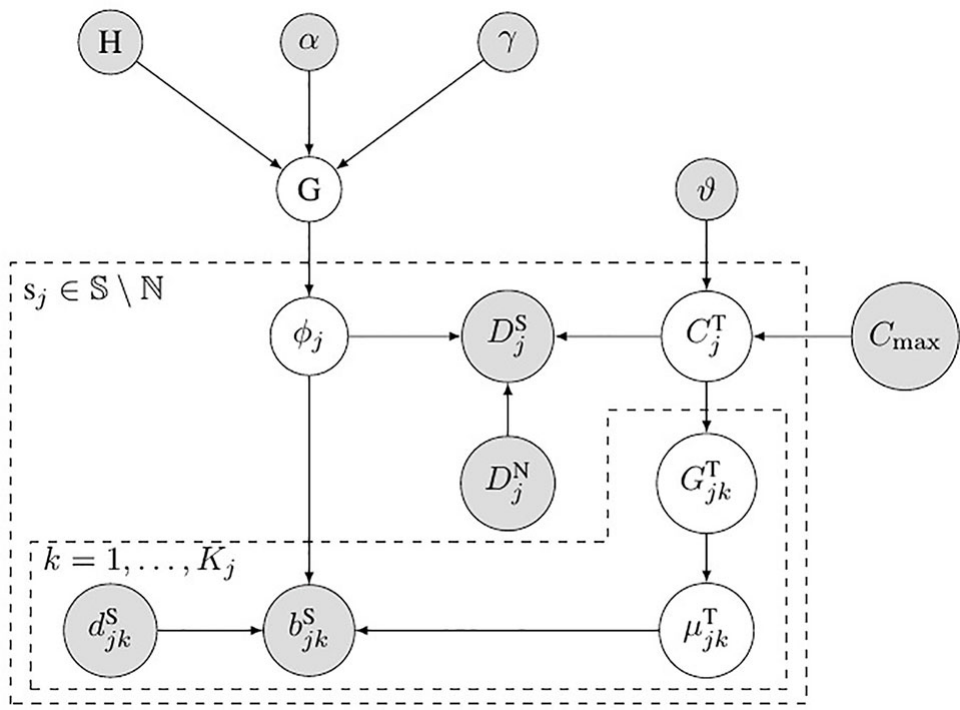
Bayesian network model for subclonal population frequency. In this figure, G denotes the tree-structured Dirichlet process; H denotes the base distribution; α and γ are the scaling parameters of G; *ϕ_j_* denotes the subclonal frequency of SCNA in segment s*_j_*; $D_{j}^{S}$ denotes the number of tumor reads mapped in segment s*_j_*, while $D_{j}^{N}$ denotes the number of normal reads mapped in segment s*_j_*; $C_{j}^{T}$ denotes the absolute copy number of SCNA in segment s*_j_*; ϑ denotes the geometric mean of the read count ratio of all the baseline segments ℕ; *C*_max_ is the maximum absolute copy number pre-defined; $G_{jk}^{T}$ denotes the tumor genotype of the *k*th heterozygous SNP loci in the *j*th segments s*_j_*; $u_{jk}^{T}$ denotes the tumor BAF of the *k*th heterozygous SNP loci in the *j*th segments s*_j_*;$b_{jk}^{S}$ and $d_{jk}^{S}$ denote the number of B-allele and the total allele at the *k*th heterozygous SNP loci in the *j*th segments s*_j_*.

**Figure 2** shows the probabilistic graphical model of SCNA’s subclonal population frequency. In this figure, *S* denotes the set of all the SCNA segments; *ℕ* denotes the set of segments that contain no SCNA. We use the same method described in Li’s study (Li and Xie, 2015) to set the probability of BAF to obey binomial distribution

(6) $$b_{jk}^{\text{S}}|d_{jk}^{\text{S}},\mu_{jk}^{\text{T}},\phi_{j}\;∼\text{Binomial}\left(d_{jk}^{\text{S}},{\hat{\bar{\mu }}}_{jk}\right),$$

where $b_{jk}^{\text{S}}$ denotes the number of tumor reads that contain B allele at the *k*th heterogeneous SNP locus and $d_{jk}^{\text{S}}$ denotes the total number of tumor reads mapped at this locus. In this figure, $G_{jk}^{\text{T}}$ denote the allele’s genotype at the *k*th heterogeneous snp locus in segment s*_j_*.

According to Equation 4, we have the expected tumor read counts mapped to segment *j*

(7) $$\lambda_{j}={\text{Φ}}^{-1}\left(\frac{{\bar{C}}_{j}}{{\bar{C}}_{i}}\times \hat{D_{i}^{\text{S}}/D_{i}^{\text{N}}}\right)\times D_{j}^{\text{N}}$$

where *Φ* ^−1^() denotes the reverse process of bias correction. Let |ℕ| denote the number of baseline segments (Li and Xie, 2015) (in which the absolute copy number $C_{j}^{\text{T}}=2$). We use the average of read count’s log ratio of all the baseline segments $\vartheta =\sqrt[{-\left|\mathbb{N}\right|}]{\prod_{{\text{s}}_{i}\in \mathbb{N}}\hat{D_{i}^{\text{S}}/D_{i}^{\text{N}}}}$ to calculate the expectation of tumor read count, and model the tumor read count as a Poisson distribution

(8) $$D_{j}^{\text{S}}|D_{j}^{\text{N}},C_{j}^{\text{T}},\phi_{j}\;∼Poisson\left(\Phi^{-1}\left(\frac{{\bar{C}}_{j}}{2}\times \vartheta \right)\times D_{j}^{\text{N}}\right)$$

It could be deduced from the first equation in Equation set 1 that ${\bar{C}}_{j}>2\Leftrightarrow C_{j}^{\text{T}}>2$. Therefore, we may conclude that $\hat{D_{j}^{\text{S}}/D_{j}^{\text{N}}}>\vartheta \Leftrightarrow C_{j}^{\text{T}}>2$, since ${\bar{C}}_{i}$ must equal 2 if *s* *_i_* contains no SCNA. We set $C_{j}^{\text{T}}$ obeys the categorical distribution

(9) $$C_{j}^{\text{T}}\;∼\text{Categorical}\left(\varsigma \left(\vartheta \right)\right),$$

where function ς (ϑ) denotes $C_{j}^{\text{T}}$‘s range; ς (ϑ) = {0, 1, 2} if $\hat{D_{j}^{\text{S}}/D_{j}^{\text{N}}}<\vartheta$; ς (ϑ) = {2, 3,…, *C*_max_} if $\hat{D_{j}^{\text{S}}/D_{j}^{\text{N}}}>\vartheta$.

The subclonal population frequency of certain mutation equals the sum of all its subpopulation frequencies (for details, refer to **Figure S1** in the **Supplementary**), and all the subpopulation frequencies in the tumor sample sums to 1. Therefore, all the subpopulation frequencies in the tumor sample obey the Dirichlet distribution, and this Dirichlet distribution obeys the tree-structured Dirichlet process (DP) (Prescott Adams et al., 2010). Suppose there are *P* subpopulations in a tumor sample; let *x*_1_,…, *x_p_* denote all the subpopulation frequencies

(10) $$x_{1},\ldots ,x_{P}\;∼\text{Dirichlet}(\alpha_{1},\ldots ,\alpha_{P}),$$

where *α*_1_,…, *α_p_* are the concentration parameters. In this paper, we set *α*_1_ = … = *α_p_* = 1, then Equation 10 is transformed into a uniform distribution of (*p* −1)-dimension simplex. Therefore, the prior probability of subclonal frequency *ϕ_j_* equals the probability of the tree structure. In **Figure 2**, *G* denotes the tree-structured DP; H denotes the base distribution; α and γ are the scaling parameters of *G*.

We use MCMC to obtain the prior distribution of *ϕ_j_* since the probability of tree-structured DP cannot be explicitly expressed. We use the slice sampling method described in Prescott’s study (Prescott Adams et al., 2010) to generate tree structure. The complete posterior probability of the subclonal population frequencies of all the SCNA segments

(11) $$\begin{array}{c} \text{Pr}\left({\left\{\phi_{j}\right\}}_{s_{j}\in S\backslash \mathbb{N}}^{}|{\left\{D_{J}^{S}\right\}}_{s_{j}\in S\backslash \mathbb{N}},{\left\{{\left\{b_{jk}^{S}\right\}}_{k=1}^{K_{j}}\right\}}_{sj\in S\backslash \mathbb{N}},T\right)\\ \propto \mathrm{Pr}({\left\{D_{J}^{S}\right\}}_{S_{j}\in S\backslash \mathbb{N}},{\left\{{\left\{b_{jk}^{S}\right\}}_{k=1}^{K_{j}}\right\}}_{S_{j}\in S\backslash \mathbb{N}}|{\left\{\phi_{j}\right\}}_{S_{j}\in S\backslash \mathbb{N}})\times \mathrm{Pr}\left({\left\{\phi_{j}\right\}}_{S_{j}\in S\backslash \mathbb{N}}\right)\\ =\prod_{N\in TC_{j}^{T}\in }\sum_{\left\{0,1...\;C_{\max}\right\}}\sum_{G_{jk}^{T}\in \zeta \left(C_{j}^{T}\right)\mu_{jk}^{T}}\sum_{\in \eta \left(G_{jk}^{T}\right)}\prod_{Sj\in N}\\ {}[\frac{1}{D_{j}^{S}!}\times {\left(\Phi^{-1}\left(\frac{{\bar{C}}_{j}}{2}\times^{|\mathbb{N}|}\sqrt{\prod_{s_{i}\in \mathbb{N}}\hat{D_{i}^{S}/D_{i}^{N}}}\right)\times D_{j}^{N}\right)}^{D_{j}^{S}}\times \\ e^{-\phi^{-1}}\left(\frac{{\bar{C}}_{j}}{2}\times \sqrt[{|\mathbb{N}}]{\prod_{s_{i}\in \mathbb{N}}\hat{D_{i}^{S}/D_{i}^{N}}}\right)\times D_{j}^{N}\times \prod_{k=1}^{K_{j}}\left(\begin{array}{c} d_{jk}^{S}\\ b_{jk}^{S} \end{array}\right)\begin{array}{c} {\hat{\bar{\mu }}}_{jk}^{b_{jk}^{S}} \end{array}{\left(1-{\hat{\bar{\mu }}}_{jk}\right)}^{\left(d_{jk}^{S}-b_{jk}^{S}\right)}]. \end{array}$$

where T denotes the tree structure, and N denotes a node in T. We select the tree structure with maximum posterior probability

(12) $$T_{\max}=\begin{array}{c} \arg\max\mathrm{Pr}\\ T^{\left(i\right)} \end{array}\left({\left\{D_{j}^{\text{S}}\right\}}_{{\text{S}}_{j}\in S\backslash \mathbb{N}},{\left\{{\left\{b_{jk}^{\text{S}}\right\}}_{k=1}^{K_{j}}\right\}}_{{\text{S}}_{j}\in S\backslash \mathbb{N}}|{\left\{\phi_{j}\right\}}_{{\text{S}}_{j}\in S\backslash \mathbb{N}}^{\left(i\right)},T^{\left(i\right)}\right),$$

where $T^{\left(i\right)}$ and ${\left\{\phi_{j}\right\}}_{{\text{s}}_{j}\in S\backslash \mathbb{N}}^{\left(i\right)}$ denote tree structure and subclonal population frequencies of the *i*th sampling process. The absolute copy number of the *i*th sampling process is

(13) $$\begin{array}{l} {\left\{C_{j}^{T}\right\}}_{S_{j}\in S\backslash \mathbb{N}}^{\left(i\right)}=\underset{N\in T^{\left(i\right)}}{\cup }\begin{array}{c} \arg\max\\ {\left\{C_{j}^{T}\right\}}_{S_{j}\in N} \end{array}\prod_{S_{j}\in \text{N}}[\frac{1}{D_{J}^{S}!}{\left({\text{Φ}}^{-1}\left(\frac{{\bar{C}}_{j}}{2}\sqrt[{|\mathbb{N}|}]{\prod_{{\text{s}}_{i}\in \mathbb{N}}\hat{D_{i}^{\text{S}}/D_{i}^{\text{N}}}}\right)\times D_{j}^{\text{N}}\right)}^{D_{j}^{\text{S}}}\times \\ e^{-{\text{Φ}}^{-1}}\left(\frac{{\bar{C}}_{j}}{2}\times \sqrt[{|\mathbb{N}|}]{\prod_{{\text{s}}_{i}\in \mathbb{N}}\hat{D_{i}^{\text{S}}/D_{i}^{\text{N}}}}\right)\times D_{j}^{\text{N}}\times \prod_{k=1}^{K_{j}}\left(\begin{array}{c} d_{jk}^{\text{S}}\\ b_{jk}^{\text{S}} \end{array}\right)\begin{array}{c} {\hat{\bar{\mu }}}_{jk}^{b_{jk}^{\text{S}}} \end{array}{\left(1-{\hat{\bar{\mu }}}_{jk}\right)}^{\left(d_{jk}^{\text{S}}-b_{jk}^{\text{S}}\right)}] \end{array},$$

where ${\left\{C_{j}^{\text{T}}\right\}}_{{\text{s}}_{j}\in S\backslash \mathbb{N}}^{\left(i\right)}$ are absolute copy numbers with the maximum posterior probability if the *i*'-th sampling process is the solution of Equation 12.

### The Pipeline for Reconstructing SCNA’s Subclonal Population-Based NGS Data

**Figure 3:**
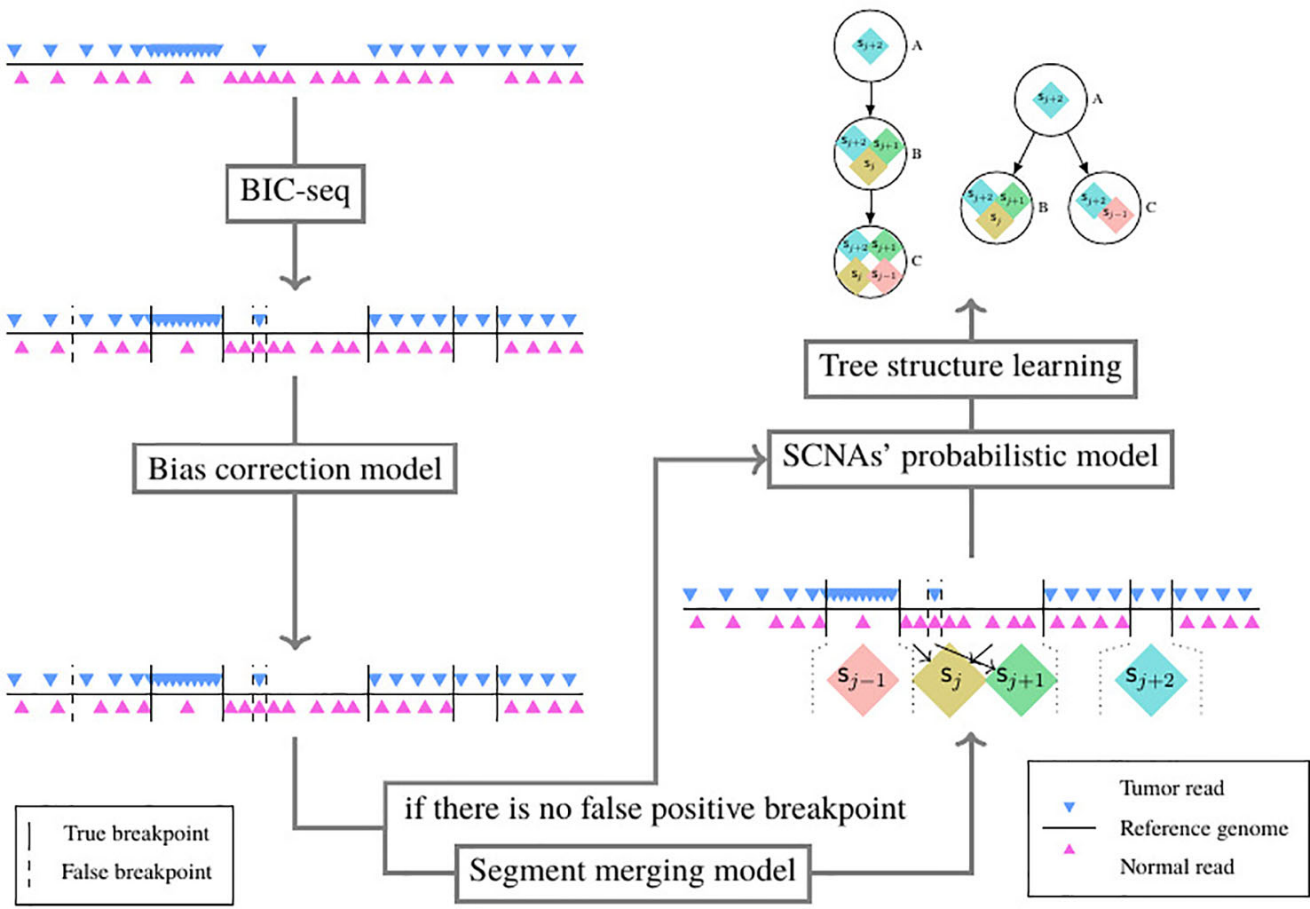
The structure of the whole NGS data-based SCNAs’ subclonal reconstruction pipeline.

As shown in **Figure 3**, the pipeline consists of five models. The tumor and its paired normal sequence alignment sequencing data in BAM format are used as input of the pipeline. The SCNA segments are detected by BIC-seq (Xi et al., 2010), then the bias of read count ratio is corrected by the correction model (Chu et al., 2017a) we previously proposed. We filter out the false positive breakpoints by the algorithm we proposed in this paper, then we use the probability model of subclonal population frequency proposed in this paper to infer the subclonal frequency of each SCNA segment. Finally, we use the tree structure learning algorithm (Prescott Adams et al., 2010) to reconstruct the SCNA’s subclonal population.

## Results

In this section, we evaluate the performance of probabilistic model on both simulated and real datasets and compare its performance with existing tools. Existing tools such as Mixclone (Li and Xie, 2015) and TheatA (Oesper et al., 2013) could not calculate the subclonal frequencies of more than three subclonal populations. Therefore, we use the simulated data, which contain more than three subclonal populations and TCGA benchmark data together to evaluate our model.

### Results From Simulated Data

**Figure 4:**
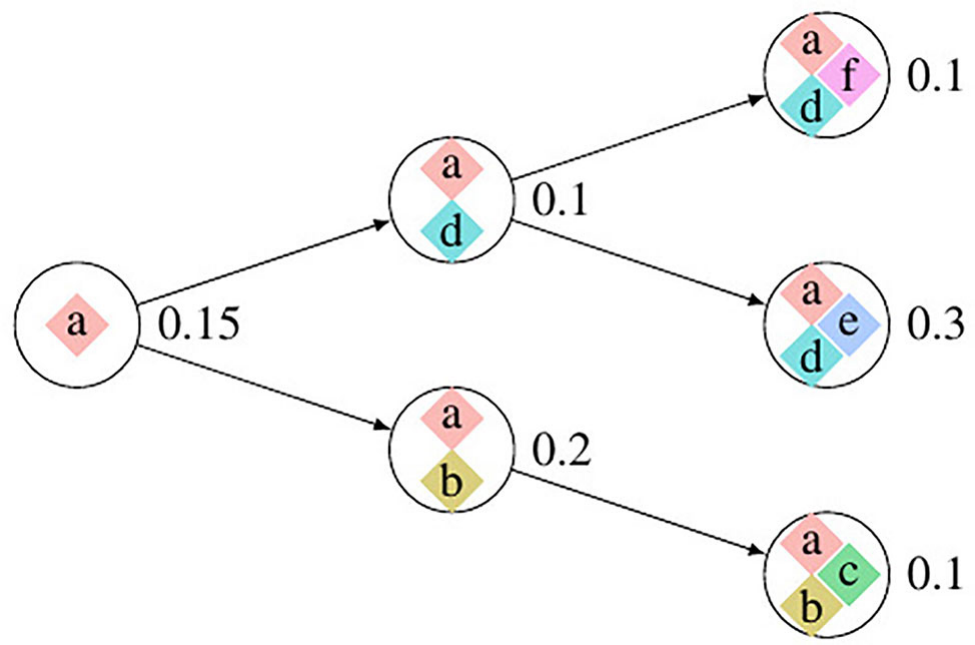
The evolution process of subclonal population in the simulation data. In this figure, each circle denotes a subpopulation; the number on the left is its frequency; each square inside the circle denotes an SCNA; each arrow points an offspring subpopulation.

We use Pysubsim-tree (Chu et al., 2017b) to simulate a tumor’s NGS read alignment data from Chromosome 21 with the evolution history configuration shown in **Figure 4** and the acquired SCNA’s configuration listed in **Table 1**. In **Figure 4**, each circle represents a subpopulation; the squares with character a, b, c, d, e, and f represent five SCNAs; the number on the right side of the circle is the frequency of the subpopulation.

**Table 1:** The SCNA’s configuration for each subpopulation of the simulation data.

SCNA	Chrom	Position	Length	$C_{j}^{T}$	*G_j_*	*ϕ_j_*
a	chr21	17478172	500000	0	Ø	0.95
b	chr21	27485802	500000	3	PPM	0.03
c	chr21	30959067	500000	4	PPPM	0.01
d	chr21	35841868	500000	5	PMMMM	0.05
e	chr21	43277023	500000	1	M	0.03
f	chr21	25056314	500000	7	MPPPPPP	0.01

**Figure 5:**
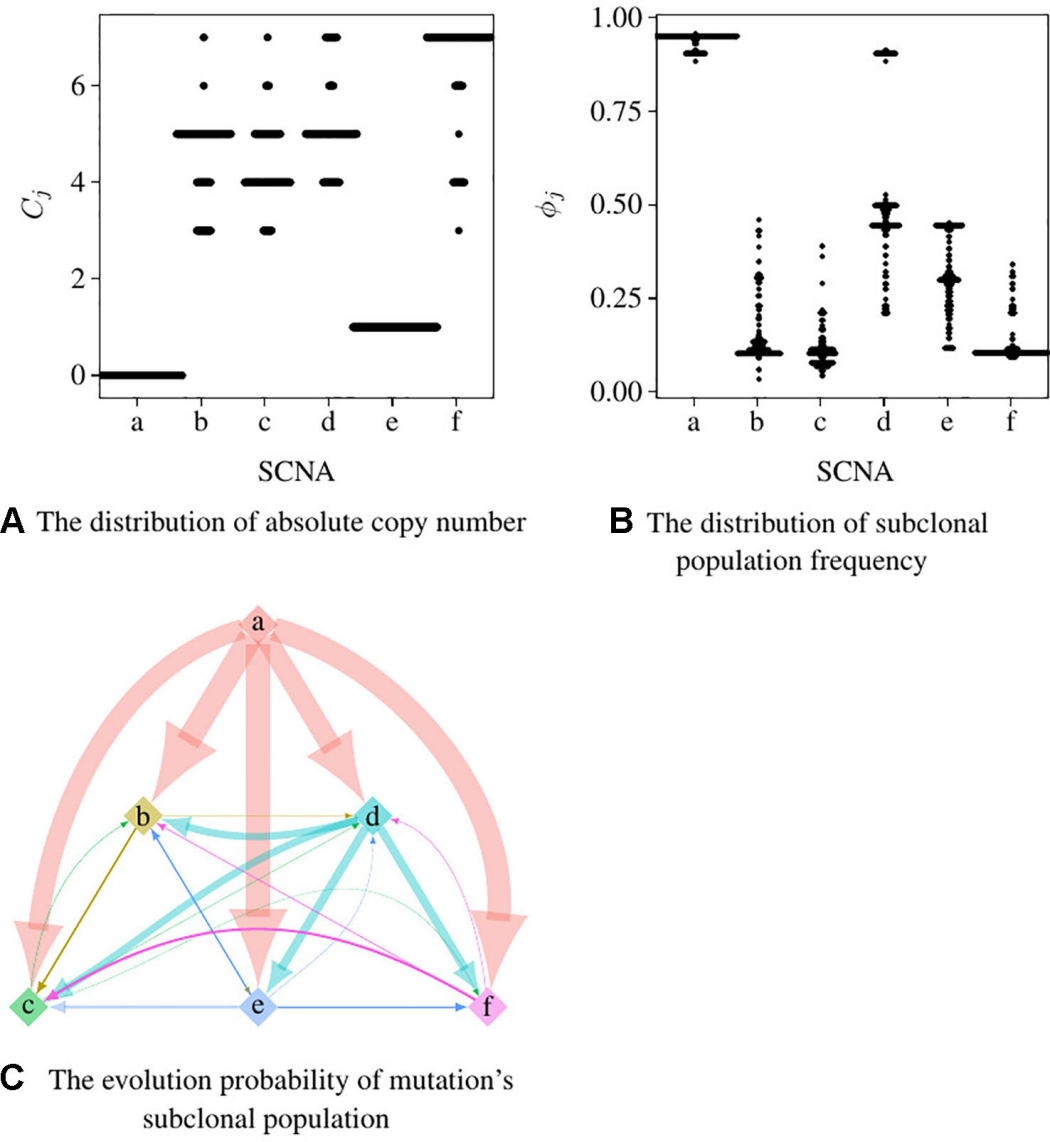
The result of subclonal reconstruction based on simulation data. **(A**, **B)** Dot-plots of the distribution of absolute copy number and subclonal frequency inferred by the 300 cycles of MCMC process. **(C)** The partial plot of the subclonal frequency.

We set the first 50 cycles of the MCMC sampling process as burn-in and use the result of the following 300 cycles to calculate the probability of the evolutionary relationship between subpopulations. We set α = 1.0, γ = 1.0, H to be the uniform distribution. **Figures 5A**, **B** are the dot-plots of the distribution of the output of subclonal population frequency model. **Figure 5C** shows the partial order plot (Jiao et al., 2014) of the evolutionary relationship obtained by the model proposed in this paper. The arrows in this figure denote the direct evolutionary relationship of the two subpopulations. The width of the arrow denotes the probability of this evolutionary relationship present in the 300 cycles of the MCMC process. Suppose ${\left\{T_{i}\right\}}_{i=1}^{I}$ denotes all the trees obtained in all the cycles of the MCMC process, $\vec{\text{ab}}$ denotes the evolutionary relationship from subpopulation a to b. Then the probability of this evolutionary relationship is

(14) $$\mathrm{Pr}\;(\vec{\mathrm{ab}})=\frac{1}{I}\left|\left\{T_{i}|\vec{\mathrm{ab}}\in T_{i},i=1,\ldots ,I\right\}\right|.$$

According to Theorem 1, a and e have only one solution of *ϕ_j_* while the others are not. The distribution of absolute copy numbers shown in **Figure 5A** is consistent with Theorem 1. The distribution of e’s subclonal frequency is quite scattered in **Figure 5B** because the small subclonal frequency and the absolute copy number of e (closed to normal) cause the coverage to decrease by 5%, which is almost the same as the noise. The subclonal frequencies of other SCNAs are highly distributed at the positions of subclonal frequencies listed in **Table 1**. Each SCNA’s absolute copy number and subclonal frequency with the maximum posterior probability are listed in **Table 2**. The subclonal frequencies of b and c are not correct because they have multiple solutions of subclonal frequencies according to Theorem 1, while the others are correct. The distribution of absolute copy number and subclonal frequency in **Figure 5** and the result listed in **Table 2** show that our SCNA probability model could correctly calculate the subclonal frequency of SCNA.

**Table 2:** The results of subclonal population frequency inferring based on simulation data.

	a	b	c	d	e	f
$C_{j}^{T}$ result	0	7	5	5	1	7
$C_{j}^{T}$ fact	0	3	4	5	1	7
*ϕ*_*j*_ result	0.950	0.106	0.075	0.501	0.304	0.106
*ϕ*_*j*_ fact	0.95	0.30	0.10	0.50	0.30	0.10

### Results From Breast Cancer Sequencing Data

We use the ngs data “HCC1954-spiked1-n25t35s40” and “HCC1954-spiked1-n25t55s20” (denoted as “n25t35s40” and “n25t55s20” for convenience) of Cancer Genome Atlas (TCGA) Benchmark 4 dataset, which is publicly available at the National Cancer Institute GDC Data Portal (https://gdc.cancer.gov/resources-tcga-users/tcga-mutation-calling-benchmark-4-files) to further validate the subclonal frequency model proposed in this paper. HCC1954 is an immortal cell line derived from an invasive ductal carcinoma of the breast diagnosed in a 61-year-old woman (Bignell et al., 2007). “G15512.HCC1954.1” is the NGS data of this cell line, which contains one subclonal population with purity 0.99; however, this data has no ground truth of absolute copy number of the SCNA regions. “HCC1954-spiked1-n25t35s40” is generated by merging 35% of “G15512.HCC1954.1” with 25% of its paired normal NGS data and 40% of “G15512.HCC1954.1” with some SCNAs randomly spiked in it. Therefore, there are two subclonal populations in the tumor sample “HCC1954-spiked1-n25t35s40,” and their subclonal frequencies are 75% and 40%, respectively. The ISA is invalid since each subclonal population contains multiple SCNAs; thus, we set the prior probability of tree structure to obey uniform distribution, and thus Equation 11 could be rewritten as follows:

(15) $$\begin{array}{c} \mathrm{Pr}\left(\phi_{j}{\left\{D_{j}^{S}\right\}}_{S_{j}\in S\backslash \mathbb{N}},{\left\{b_{jk}^{S}\right\}}_{k=1}^{k_{j}},T\right)\propto \mathrm{Pr}\left({\left\{D_{j}^{S}\right\}}_{S_{j}\in S\backslash \mathbb{N}}{\left\{b_{jk}^{S}\right\}}_{k=1}^{k_{j}},T|\phi_{j}\right)\\ =\prod_{s_{j}\in S\backslash \mathbb{N}C_{j}^{T}\in }\sum_{\left\{0,1\ldots C_{\max}\right\}}|[\frac{1}{D_{j}^{S}!}\times {\left(\Phi^{-1}\left(\frac{{\bar{C}}_{j}}{2}\times \sqrt[{|\mathbb{N}|}]{\prod_{s_{i}\in \mathbb{N}}\hat{D_{i}^{S}/D_{i}^{N}}}\right)\times D_{j}^{N}\right)}^{D_{j}^{S}}\times \\ e^{-\Phi^{-1}}\left(\sqrt[{|\mathbb{N}|}]{\prod_{s_{i}\in \mathbb{N}}\hat{D_{i}^{S}/D_{i}^{N}}}\right)\times D_{j}^{N}\times \\ \prod_{k=1}^{K_{j}}\sum_{G_{jk}^{T}\in \zeta \left(C_{j}^{T}\right)\mu_{jk\in \;\eta }^{T}}\sum_{\left(G_{jk}^{T}\right)}\left(\begin{array}{c} d_{jk}^{S}\\ b_{jk}^{S} \end{array}\right)\begin{array}{c} {\hat{\bar{\mu }}}_{jk}^{b_{jk}^{S}} \end{array}{\left(1-\hat{\bar{\mu }}\right)}^{\left(d_{jk}^{S}-b_{jk}^{S}\right)}] \end{array}$$

**Figure 6:**
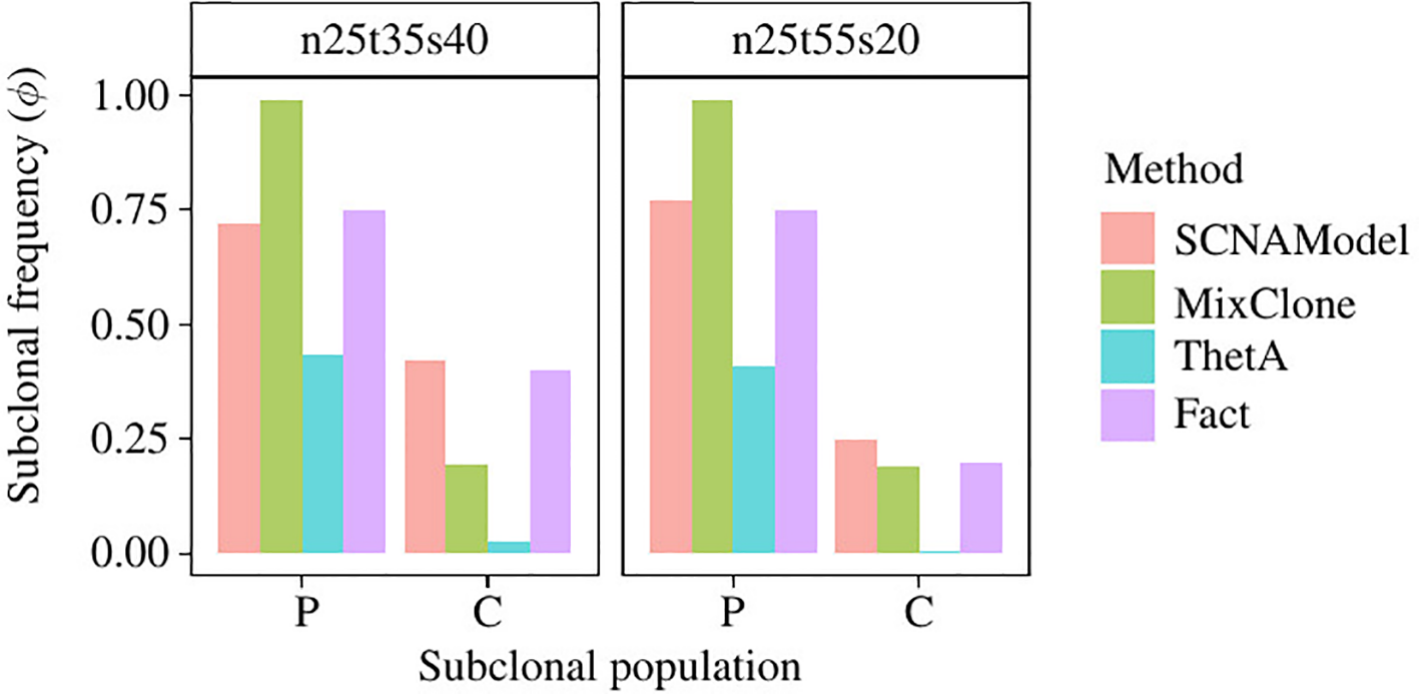
The subclonal proportion of SCNAs in HCC1954 data. In this figure, SCNAModel is the subclonal frequency inferring model proposed in this paper.

**Figure 6** shows the subclonal frequencies obtained by the model proposed in this paper. In this figure, “P” denotes the parent subclonal population (subclonal frequency 75%) and “C” denotes the child subclonal population (subclonal frequency 40%). As shown in **Figure 6**, the subclonal frequencies of these two population obtained by the model proposed in this paper are 72% and 42% for sample “n25t35s40” and 77% and 25% for sample “n25t55s20,” which are the most closed to the fact in comparison with MixClone and ThetA.

## Discussion

**Figure 7:**
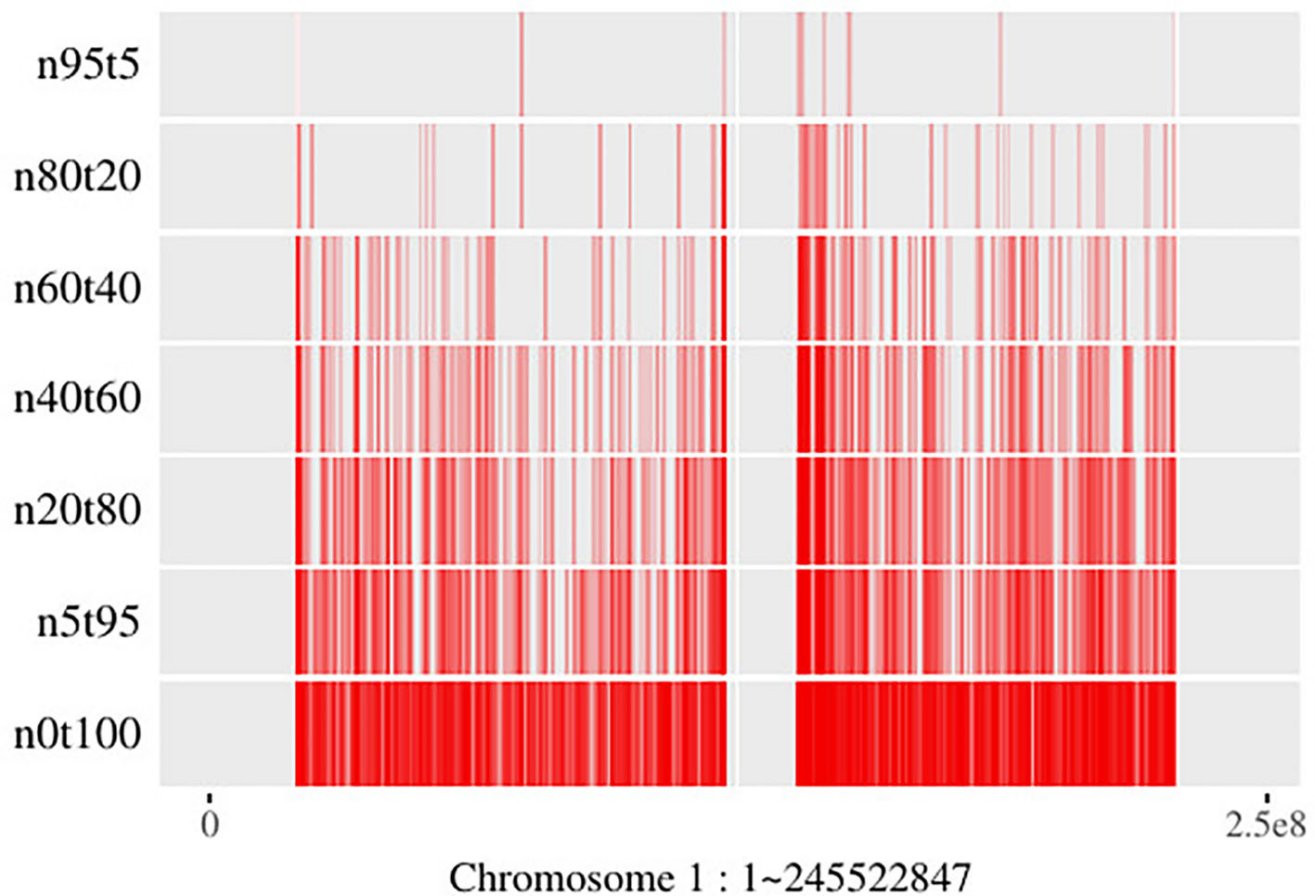
Breakpoints distribution on chromosome 1 of mixed “HCC1954” samples. Here the “n5t95” to “n95t5” respectively denote the tumor sample from “HCC1954.mix1.n5t95” to “HCC1954.mix1.n95t5.” “n0t100” denotes the tumor sample; “HCC1954” contains no normal contamination. Each of these samples contains one tumor subclone. All the breakpoints are obtained by BIC-seq (Xi et al., 2010).

Generally, SCNAs with larger subclonal population frequency could relatively be more precisely located. However, due to the twice sequencing procedures of tumor and its paired normal, the read information of the genomic regions with the same copy number in tumor sample is not exactly the same as its paired normal’s. Moreover, the lower read coverage of NGS makes the noise/error more likely to be mistaken for an SCNA. As shown in **Figure 7**, the number of SCNA breakpoints obtained by SCNA detection tool is proportional to the subclonal population frequency. If there exists a large proportion of false negative breakpoints, it will cause the read count in the segments incapable to reveal the copy number property, then it will affect all the read count-based SCNA analysis tools. On the other hand, if there exists a large proportion of false positive breakpoints, the segment clustering step of filtering out the false positive breakpoints could reduce the data size and make the read count information more robust to noise by merging the SCNA segments with the same absolute copy number and subclonal population frequency. As shown in Theorem 1, the SCNA segments with the same RCR and average B-allele frequency are indistinguishable to the NGS-based SCNA analysis tools. Merging two non-adjacent SCNA segments with the same NGS properties could not affect the result of the NGS-based SCNA analysis tools.

Tree-Structured Stick Breaking (TSSB) process (Prescott Adams et al., 2010) could learn the tree structure of the hierarchical data. A tree structure space could be generated by intertwining two DP; then as described in Prescott’s paper (Prescott Adams et al., 2010), one can imagine throwing a dart (data) on the tree space and considering which node the dart hits. If we know subclonal number *L* in advance, then we could generate the tree structure in two steps. Step 1: generate a tree using all the data; Step 2: sort nodes by the sum of the size of the genome region hit, then find out the top *L* nodes and throw the rest of the darts (data not in the *L* nodes) into these *L* nodes randomly. **Figure 7** shows that subclonal frequency affects the number of breakpoints; thus, there might present false positive or false negative breakpoints in the result of the SCNA detection tool. The false positive breakpoints could be filtered out by the algorithm in this paper. Even if there exist false breakpoints, the redundant data that contains the same SCNA might hit the same node in the tree space generated by the TSSB process. Thus, the redundant data affects the time and space consumption, but could not affect the result of subclonal reconstruction theoretically.

## Conclusion

In this paper, we first perform a mathematical analysis of the solution space of SCNA’s subclonal frequency. Then based on the mathematical analysis, we propose an algorithm to filter out the false breakpoints and we construct a new probability model to reconstruct SCNA’s subclonal population, which incorporates the algorithms of RCR bias correction we previously proposed. We use the tree-structured stick breaking DP (Prescott Adams et al., 2010) to generate the tree structure space of tumor’s evolutionary history. In the probability model, the BAF of the heterozygous SNP locus in the SCNA segment is modeled as a binomial distribution and the read depth of tumor sampling data is modeled as a Poisson distribution with respect to the potential bias in RCR. We generate the distribution of subclonal frequency from the distribution of subpopulation frequency, which is drawn from the tree structure space. By stringing the model with the false breakpoint filtering algorithm, we construct a whole SCNA’s subclonal population reconstruction pipeline, which is capable of inferring SCNA’s absolute copy number and its subclonal population frequency and its evolutionary process while there are a lot of false positive SCNA breakpoints and the RCR presents bias. The results show that the model proposed in this paper could more accurately estimate the absolute copy number of SCNA segments and their subclonal population frequencies in comparison with existing methods both on simulated data and TCGA data.

## Data Availability Statement

Publicly available datasets were analyzed in this study. This data can be found here: https://gdc.cancer.gov/resources-tcga-users/tcga-mutation-calling-benchmark-4-files.

## Author Contributions

YC: Coming up with the theories and all the mathematical equations in this paper and implemented the initial version of P-SCNAClonal, the initial version of this paper. CN: Debugging of the initial version of P-SCNAClonal, experiments and result collecting, completed this paper with the result section. YW: Providing the basic idea and funding support.

## Funding

This work was supported by funding from the National Key R&D Program of China (No: 2016YFC1202302 and 2017YFSF090117) and the National Nature Science Foundation of China (Grant No. 61822108 and 61571152).

## Conflict of Interest

The authors declare that the research was conducted in the absence of any commercial or financial relationships that could be construed as a potential conflict of interest.
