# Supplementary material for: A Pipeline for Reconstructing Somatic Copy Number Alternation’s Subclonal Population-Based Next-Generation Sequencing Data
Source: Front Genet. 2020 Feb 27;10:1374. doi: 10.3389/fgene.2019.01374 (PMC7058119; doi:10.3389/fgene.2019.01374)
Supplement: Supplementary file 1 [file DataSheet_1.pdf]

Supplementary Material

CONTENTS

1 Background of tree-structure sampling 1
2 How and why the bias of read count ratio affects the SCNA analysis/subclonal reconstruction 2
3 The solution space of C_j^{T} and ϕ_j 3
4 The validation for false breakpoints filtering algorithm (FBFA) 7

1 BACKGROUND OF TREE-STRUCTURE SAMPLING

Many data are naturally modeled by an unobserved hierarchical structure. Ryan[8] propose a flexible nonparametric prior over unknown data hierarchies. The approach uses nested stick-breaking processes to allow for trees of unbounded width and depth, where data can live at any node and are infinitely exchangeable. The model can be viewed as providing infinite mixtures where the components have a dependency structure corresponding to an evolutionary diffusion down a tree. By using a stick-breaking approach, the users can apply Markov chain Monte Carlo methods based on slice sampling to perform Bayesian inference and simulate from the posterior distribution on trees.

Let $\boldsymbol{\epsilon} = (\epsilon_1, \ldots, \epsilon_p)$ denote a sequence of positive integers used to index the nodes of the tree. Let $\boldsymbol{\epsilon} = \kappa$ denote the zero-length string, i.e., the root of the tree. Let $|\boldsymbol{\epsilon}|$ indicate the length of the sequence $\boldsymbol{\epsilon}$ and therefore the depth of node $\boldsymbol{\epsilon}$. Let $\boldsymbol{\epsilon}\epsilon_i$ denote the sequence formed by appending ϵ_i to $\boldsymbol{\epsilon}$. The children of node $\boldsymbol{\epsilon}$ is the set $\{\boldsymbol{\epsilon}\epsilon_i : \epsilon_i \in 1, 2, \ldots\}$ and let the ancestors of $\boldsymbol{\epsilon}$ be denoted by the set $\{\boldsymbol{\epsilon}' : \boldsymbol{\epsilon}' \prec \boldsymbol{\epsilon}\}$. As shown in Figure S1, the interleaved, two-layered stick-breaking construction is as follows:

$$\begin{aligned}
&\nu_{\boldsymbol{\epsilon}} \sim \mathrm{Beta}(1, \alpha(|\boldsymbol{\epsilon}|)); \psi_{\boldsymbol{\epsilon}} \sim \mathrm{Beta}(1, \gamma); \pi_{\varnothing} = \nu_{\varnothing};\\
&\pi_{\boldsymbol{\epsilon}} = \nu_{\boldsymbol{\epsilon}}\varphi_{\boldsymbol{\epsilon}} \prod_{\boldsymbol{\epsilon}' \prec \boldsymbol{\epsilon}} \varphi_{\boldsymbol{\epsilon}'}(1 - \nu_{\boldsymbol{\epsilon}'}); \varphi_{\boldsymbol{\epsilon}\epsilon_i} = \psi_{\boldsymbol{\epsilon}\epsilon_i} \prod_{j=1}^{\epsilon_{i-1}} (1 - \phi_{\boldsymbol{\epsilon}_j}).
\end{aligned} \tag{S1}$$

The $\nu_{\boldsymbol{\epsilon}}$ and $(1 - \nu_{\boldsymbol{\epsilon}})$ determine the amount of mass allocated to $\boldsymbol{\epsilon}$ and its descendants respectively, whereas $\{\psi_{\boldsymbol{\epsilon}}\}$ determines the probability of a particular sequence of children. The construction ensures that the mixing weights $\{\pi_{\boldsymbol{\epsilon}}\}$ sum to one. The parameters α and γ control the height and the width of the tree respectively. Note that the concentration parameter $\alpha(\cdot)$ is a function of the depth of the tree $(\alpha(\cdot) : \mathbb{N} \to \mathbb{R}^{+})$ and is defined to be $\alpha(j) = \lambda^j \alpha_0$ with $\alpha_0 > 0$ and $\lambda \in (0, 1]$[8]. The generative process for infinite mixture models is

$$\boldsymbol{\pi} \sim \mathsf{GEM}(\alpha); z_i \sim \mathsf{Multinomial}(\boldsymbol{\pi}); \phi_k \sim \mathsf{H}; x_i \sim F(\phi_{z_i}), \tag{S2}$$

where GEM denotes the , $z_i \in \{1, \ldots, K\}$ is the cluster assignment variable, H is the prior distribution from which the component parameters $\{\phi_k\}$ are drawn, $F(\phi)$ is the component distribution parameterized by ϕ.

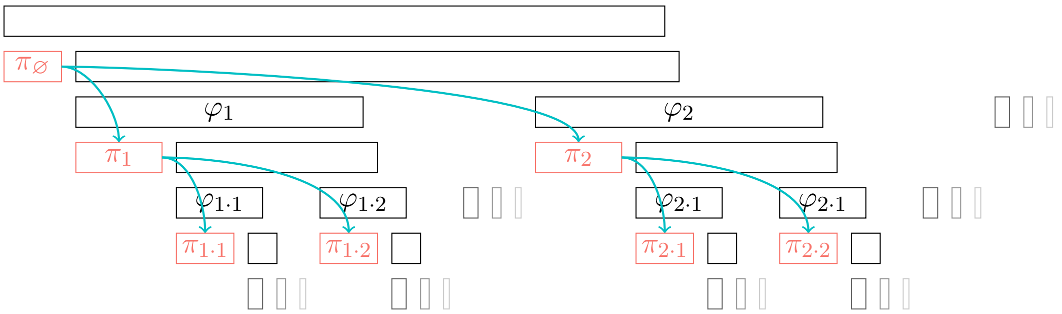

Figure S1: Hierarchical dirichlet process tree-structured stick-breaking procedure

2 HOW AND WHY THE BIAS OF READ COUNT RATIO AFFECTS THE SCNA ANALYSIS/SUBCLONAL RECONSTRUCTION

Let coefficient θ_j denote the effect of mappability and genomic length of segment j, $\bar{C}_j$ denote the average copy number of segment j, λ_j denote the expected read counts, and let D_j^N denote the read counts of segment j in matched normal genome, then for segment i and segment j, existing SCNA based tumor subclonal populations inferring tools [5, 7] assume that $\lambda_i/\lambda_j = \bar{C}_i\theta_i/\bar{C}_j\theta_j$, and $\theta_i/\theta_j = D_i^N/D_j^N$, then

$$\frac{D_i^S}{D_j^S} = \frac{\lambda_i}{\lambda_j} = \frac{\bar{C}_i\theta_i}{\bar{C}_j\theta_j} = \frac{\bar{C}_i}{\bar{C}_j} * \frac{D_i^N}{D_j^N}. \tag{S3}$$

Figure S2 shows the two normal libraries from the same normal sample, and there is a crossover point of the two loess lines. Here we suppose the normal Lib 2 is a tumor sample has no variations, and normal Lib 1 is its paired normal sample. According to Equation S3,

$$\frac{D_i^{Lib2}}{D_j^{Lib2}} = \frac{\lambda_i}{\lambda_j} = \frac{\bar{C}_i\theta_i}{\bar{C}_j\theta_j} = \frac{2}{2} * \frac{D_i^{Lib1}}{D_j^{Lib1}} = \frac{D_i^{Lib1}}{D_j^{Lib1}}, \tag{S4}$$

If j is the crossover point, we have $D_i^{Lib2} = D_i^{Lib1}$, which means the two loess lines should overlap each other. This demonstrates that the GC bias is different in the tumor and its paired normal sample.

By increasing the window size to 5000bp (Figure S3c) or even larger at SCNA level (Figure S3b), the 2D plot between GC content and tumor-normal coverage ratio clearly clustered into multiple stripes. It is noted that the relationship is pretty linear between GC content and log ratio of tumor-normal coverage on SCNAs (Figure S3a) and we show that slopes of linear relation vary across tumors (Figure S1). We also show that the gaps between the stripes in Figure S3a are proportional to the subclonal populations (as shown in the sub-figures in the first column of Figure **??**). The SCNA segments which are clustered into the same stripe, present the symmetrical pattern of B allele frequency (BAF) density on the heterozygous allele loci of paired normal sample (Figure S3e), which reveals that these SCNA segments in the same stripe contain the same copy number(refer to supplementary 3.3.2 for detail proof). While using the ratio of read counts of SCNA segments to get the precise subclonal population of each SCNA, it needs to correct the GC bias of the gap first.

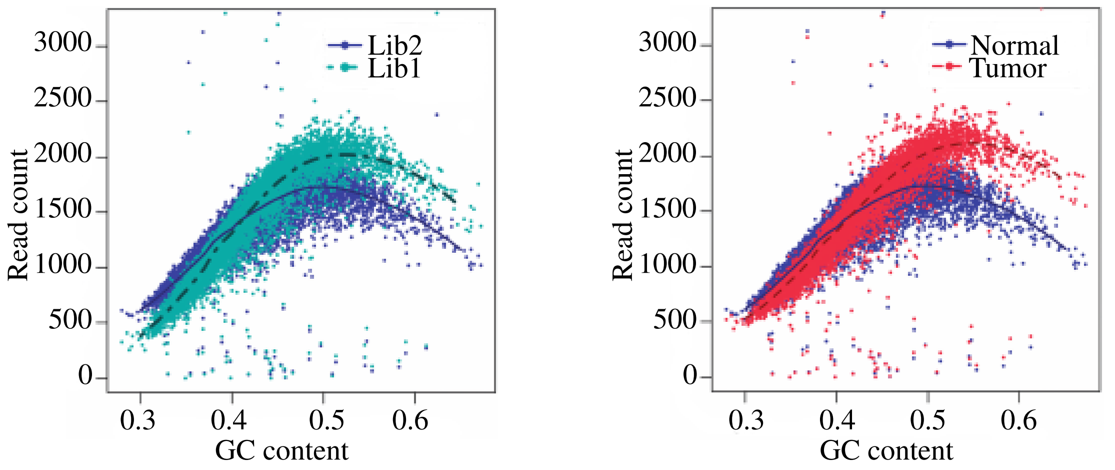

a) Two different DNA library from normal sample b) Tumor sample and its paired normal

Figure S2: GC curves (10 kb bins). Observed fragment counts and loess lines plotted against GC of two libraries from the same normal sample TCGA-13-0723.Bins were randomly sampled from chromosome 1. This Figure is drawn by Benjamini et. al[1].

3 THE SOLUTION SPACE OF C_J^{T} AND ϕ_J

$$\begin{cases} \bar{C}_j = \phi_j * C_j^{\mathrm{T}} + (1-\phi_j) * 2, \\ \bar{C}_j = \dfrac{1}{\bar{\mu}_{jk}} \left[\phi_j * C_j^{\mathrm{T}} * \mu_{jk}^{\mathrm{T}} + (1-\phi_j) * 2 * \dfrac{1}{2} \right], \; k = 1, \ldots, K_j. \end{cases} \tag{S5}$$

We calculate $\hat{\bar{\mu}}_{jk}^{\mathrm{T}}$ by following transformation

$$\hat{\bar{\mu}}_{jk}^{\mathrm{T}} = \frac{1}{2} - \left| \bar{\mu}_{jk}^{\mathrm{T}} - \frac{1}{2} \right|, \tag{S6}$$

here $0 \leqslant \hat{\bar{\mu}}_{jk}^{\mathrm{T}} \leqslant \frac{1}{2}$. Substituting into the second Equation in Equation set S5 gives $0 \leqslant \hat{\mu}_{jk}^{\mathrm{T}} \leqslant \frac{1}{2}$, then we have

$$\hat{\mu}_{jk}^{\mathrm{T}} \in \left\{ \frac{(C_j^{\mathrm{T}} - i)/2}{C_j^{\mathrm{T}}} \right\}, \tag{S7}$$

where $i = 1, 3, \ldots, C_j^{\mathrm{T}}$ if C_j^{T} is odd; $i = 0, 2, \ldots, C_j^{\mathrm{T}}$ if C_j^{T} is even.

THEOREM 1. *Given $\bar{C}_j$ and $\{\hat{\bar{\mu}}_{jk}\}_{k=1}^{K_{s_j}}$ and let $\xi = \frac{C_j^{\mathrm{T}} \hat{\mu}_{jk}^{\mathrm{T}} - 1}{C_j^{\mathrm{T}} - 2}$, we have the following conclusions*

1. *If $\bar{C}_j < 2$, there is only one solution ϕ_j in Equation set S5.*
2. *If $\bar{C}_j > 2$ and $\bar{C}_j < \frac{1}{\hat{\bar{\mu}}_{jk}}$ there is only one solution of ϕ_j in Equation set S5.*
3. *If $\bar{C}_j > 2$ and $\bar{C}_j \geqslant \frac{1}{\hat{\bar{\mu}}_{jk}}$, there are infinite solutions of ϕ_j in Equation set S5.*
4. *If $\bar{C}_j > 2$ and $\bar{C}_j \geqslant \frac{1}{\hat{\bar{\mu}}_{jk}}$, there are multiple solutions of ϕ_j in Equation set S5 on the curves of the family of function $\hat{\bar{\mu}}_{jk} = \xi \left(1 - \frac{2}{\bar{C}_j}\right) + \frac{1}{\bar{C}_j}$, under the restriction of maximum absolute copy number $C_{\max}$.*

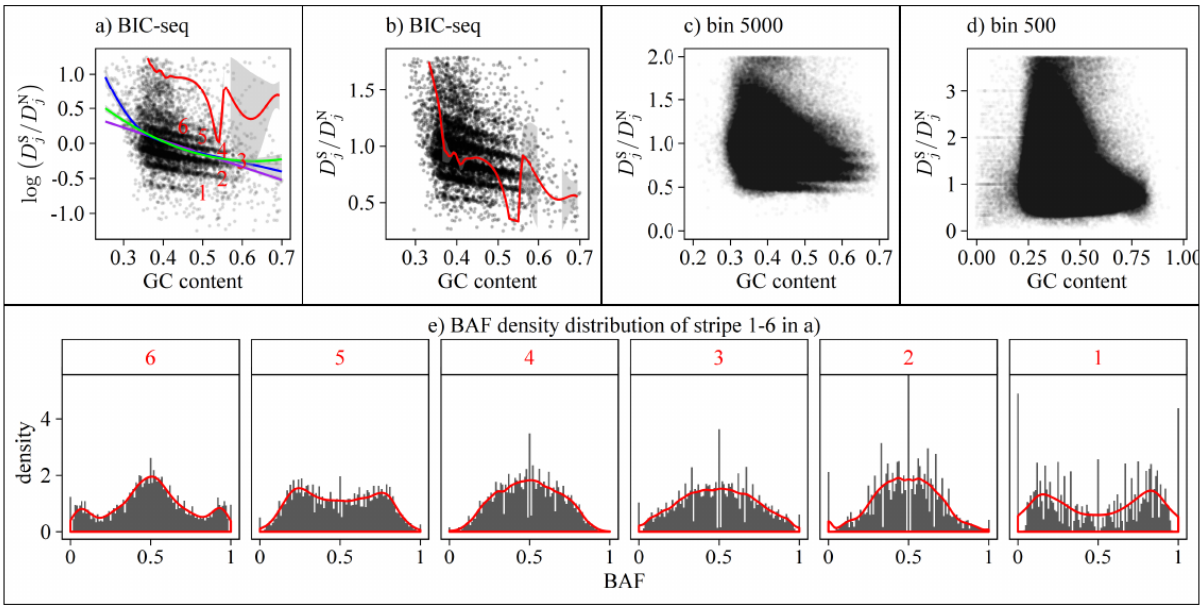

Figure S3: GC bias of WGS data of tumor-normal paired sample HCC1954.mix1.n20t80 of TCGA mutation calling benchmark 4. Let D^S and D^N respectively denote the read counts of the segment of tumor and normal samples . (a) The GC bias of the Log ratio of tumor and normal read counts of the SCNA segments. The purple and blue lines are linear regression and loess regression lines respectively. (b) The GC bias of the ratio of tumor and normal read counts of the SCNA segments. The red line are drawn by the loess regression model with a quadratic polynomial function, which is used to rectify the distribution of the ratio D^S/D^N in the state-of-art GC correction method [4]. (c) The GC bias of the ratio of tumor and normal read counts of the 5000 bp bin. Since the majority (81 %) of CNV calls are between 1 kb and 100 kb [6], most of 5000 bp bins spans only one SCNA. This sub-figure shows most SCNAs clustered clearly into multiple strips. (d) The GC bias of the ratio of tumor and normal read counts of the 500 bp bin. (e) The distribution of B-allele frequency (BAF) of stripe 1–6 in Figure 1a. The SCNA segments are obtained by BIC-seq [9].

PROOF. We now use counter-evidence to prove this theorem. Suppose there are two somatic copy number alternation (SCNA) segments $\mathrm{s}_{j'}$ and $\mathrm{s}_{j''}$ and they have the same observation, that means

$$\begin{cases} \bar{C}_{j'} = \bar{C}_{j''}, \\ \hat{\bar{\mu}}_{j'k'} = \hat{\bar{\mu}}_{j''k''} \end{cases} \tag{S8}$$

and they do have different ϕ_j and $\phi_{j'}$where $k' \in \{1, \ldots, K_{j'}\}$, $k'' \in \{1, \ldots, K_{j''}\}$.

1. If $\bar{C}_{j'} = \bar{C}_{j''} < 2$, then according to Equation set S5, we have $0 < \phi_j < 1$ and $C^{\mathrm{T}}_{j'} < 2$ and $C^{\mathrm{T}}_{j''} < 2$. Thus, it must be the case that one of $C^{\mathrm{T}}_{j'}$ and $C^{\mathrm{T}}_{j''}$ equals 1 and the other is equal to 0, so as to make $\phi_{j'} \neq \phi_{j''}$. Suppose $C^{\mathrm{T}}_{j'} = 0$, $C^{\mathrm{T}}_{j''} = 1$, we have $\hat{\bar{\mu}}^{\mathrm{T}}_{j'k'} = \frac{1}{2}$ by Equation S7 and by Equation set S5,

$$\hat{\bar{\mu}}_{j'k'} = \frac{\phi_{j'} * C^{\mathrm{T}}_{j'} * \hat{\mu}^{\mathrm{T}}_{j'k'} + (1-\phi_{j'}) * 2 * \frac{1}{2}}{\phi_{j'} * C^{\mathrm{T}}_{j'} + (1-\phi_{j'}) * 2} = \frac{(1-\phi_j) * 2 * \frac{1}{2}}{(1-\phi_j) * 2} = \frac{1}{2}, \tag{S9}$$

$$\hat{\bar{\mu}}_{j''k''} = \frac{\phi_{j''} * C^{\mathrm{T}}_{j''} * \hat{\mu}^{\mathrm{T}}_{j''k''} + (1-\phi_{j''}) * 2 * \frac{1}{2}}{\phi_{j''} * C^{\mathrm{T}}_{j''} + (1-\phi_{j''}) * 2} = \frac{1-\phi_{j''}}{2-\phi_{j''}}. \tag{S10}$$

It can be easily seen that $\hat{\bar{\mu}}_{j'k'} \neq \hat{\bar{\mu}}_{j''k''}$ because subclonal population frequency $\phi_{j''} = 0$ means there is no SCNA mutation in $\mathsf{s}_{j''}$ ($\bar{C}_{j''} = 2$), which contradicts the assumption expressed in Equation S8. Thus there is only one solution of ϕ_j, if $\bar{C}_{j'} = \bar{C}_{j''} < 2$.

2. Considering Equation set S5 and Equation set S8, we have the relationship between the unknown variables in Equation set S5 and they satisfy the following Equation set

$$\begin{cases} \dfrac{C^{\mathrm{T}}_{j'} - 2}{C^{\mathrm{T}}_{j''} - 2} = \dfrac{\phi_{j''}}{\phi_{j'}} \\ \dfrac{C^{\mathrm{T}}_{j'}\hat{\mu}^{\mathrm{T}}_{j'k'} - 1}{C^{\mathrm{T}}_{j'} - 2} = \dfrac{C^{\mathrm{T}}_{j''}\hat{\mu}^{\mathrm{T}}_{j''k''} - 1}{C^{\mathrm{T}}_{j''} - 2} \end{cases}. \tag{S11}$$

Given $\bar{C}_j < \frac{1}{\hat{\bar{\mu}}_{jk}}$ and equation set S5, we can do the following equivalent transformation

$$\begin{aligned} \bar{C}_j < \frac{1}{\hat{\bar{\mu}}_{jk}} &\Leftrightarrow \phi_j * C^{\mathrm{T}}_j * \hat{\mu}^{\mathrm{T}}_{jk} + (1-\phi_j) * 2 * \frac{1}{2} < 1 \\ &\Leftrightarrow (C^{\mathrm{T}}_j * \hat{\mu}^{\mathrm{T}}_{jk} - 1) * \phi_j < 0 \\ &\Leftrightarrow \hat{\mu}^{\mathrm{T}}_{jk} < \frac{1}{C^{\mathrm{T}}_j}. \end{aligned} \tag{S12}$$

Considering equation S7, only $\hat{\mu}^{\mathrm{T}}_{jk} = 0$ makes Equation S12 true. If $\hat{\mu^{\mathrm{T}}_{jk}} = 0$, according to the second equation in the Equation set S11, we have $C^{\mathrm{T}}_{j'} = C^{\mathrm{T}}_j$. If $C^{\mathrm{T}}_{j'} = C^{\mathrm{T}}_j$, then $\phi_{j'} = \phi_j$ according to the first equation of equation set S11. Therefore, there is only one solution to the Equation set S5 if $\bar{C}_j > 2$ and $\bar{C}_j < \frac{1}{\hat{\bar{\mu}}_{jk}}$.

3. Using the same method above, we have

$$\bar{C}_j \geqslant \frac{1}{\hat{\bar{\mu}}_{jk}} \Leftrightarrow \hat{\mu}^{\mathrm{T}}_{jk} \geqslant \frac{1}{C^{\mathrm{T}}_j}. \tag{S13}$$

According to EquationS7, let a be a non-negative integer and $a \in \{1, ..., \frac{C^{\mathrm{T}}_{j'} - i'}{2}\}$ (if $C^{\mathrm{T}}_{j'}$ is odd then $i' = 1, 3, ..., C^{\mathrm{T}}_{j'}$ and if $C^{\mathrm{T}}_{j'}$ is even, $i = 0, 2, ..., C^{\mathrm{T}}_{j'}$). Now $\frac{a}{C^{\mathrm{T}}_{j'}}$ and $\hat{\mu}^{\mathrm{T}}_{j'k'}$ shares the same range, thus we set $\hat{\mu}^{\mathrm{T}}_{j'k'} = \frac{a}{C^{\mathrm{T}}_{j'}}$.

From the first equation in Equation set S5 we can see that given $\bar{C}_j$, the solution ϕ_j and the variable C^{T}_j are one-to-one corresponded. This means if we can prove there exists countless reasonable $C^{\mathrm{T}}_{j''}$ and $\hat{\mu^{\mathrm{T}}_{j''k''}}$ that satisfy the equation set S11, then we can prove there exists infinite solution ϕ according to the Equation set S5.

For any $x \in \mathbb{N}^+$ let $b = (x+1)(a-1)+1$,

$$\hat{\mu}^{\mathrm{T}}_{j''k''} = \frac{b}{C^{\mathrm{T}}_{j''}} = \frac{(x+1)(a-1)+1}{C^{\mathrm{T}}_{j''}}, \tag{S14}$$

$$C^{\mathrm{T}}_{j''} = (x+1)(C^{\mathrm{T}}_{j'} - 2) + 2, \tag{S15}$$

from the known conditions we know that $b \geq a$, $C^{\mathrm{T}}_{j''} > C^{\mathrm{T}}_{j'}$ and both b and $C^{\mathrm{T}}_{j''}$ are positive integers. From the following procedure we show that $\hat{\mu}^{\mathrm{T}}_{j''k''}$ and the genotype of $C^{\mathrm{T}}_{j''}$ is of one-to-one correspond.

$$\begin{aligned} & a && \leqslant \frac{1}{2}C^{\mathrm{T}}_{j'}. \\ \Leftrightarrow\quad & (x+1)(a-1) && \leqslant \frac{1}{2}(x+1)(C^{\mathrm{T}}_{j'}-2) \\ \Leftrightarrow\quad & (x+1)(a-1)+1 && \leqslant \frac{1}{2}((x+1)(C^{\mathrm{T}}_{j'}-2)+2) \\ \Leftrightarrow\quad & b && \leqslant \frac{1}{2}C^{\mathrm{T}}_{j''}. \end{aligned} \tag{S16}$$

We bring equationS14 and equationS15 to Equation set S11 we now have

$$\begin{aligned} & \frac{C^{\mathrm{T}}_{j'}\hat{\mu}^{\mathrm{T}}_{j'k'}-1}{C^{\mathrm{T}}_{j'}-2} && = \frac{C^{\mathrm{T}}_{j''}\hat{\mu}^{\mathrm{T}}_{j''k''}-1}{C^{\mathrm{T}}_{j''}-2} \\ \Leftrightarrow\quad & \frac{a-1}{C^{\mathrm{T}}_{j'}-2} && = \frac{(x+1)(a-1)+1-1}{(x+1)(C^{\mathrm{T}}_{j'}-2)+2-2} \\ \Leftrightarrow\quad & \frac{a-1}{C^{\mathrm{T}}_{j'}-2} && = \frac{a-1}{C^{\mathrm{T}}_{j'}-2}, \end{aligned} \tag{S17}$$

note that $\phi_{j'} \neq \phi_{j''}$ and

$$\begin{aligned} \frac{\phi_{j''}}{\phi_{j'}} &= \frac{C^{\mathrm{T}}_{j'}-2}{C^{\mathrm{T}}_{j''}-2} \\ &= \frac{C^{\mathrm{T}}_{j'}-2}{(x+1)(C^{\mathrm{T}}_{j'}-2)+2-2} \\ &= \frac{1}{x+1}. \end{aligned} \tag{S18}$$

Thus the solution ϕ_j is also infinite, because the range of x is infinite.

4. The second equation in Equation set S5 could be transformed into following equation

$$\hat{\bar{\mu}}_{jk} = \frac{C^{\mathrm{T}}_{j}\hat{\mu}^{\mathrm{T}}_{jk}-1}{C^{\mathrm{T}}_{j}-2}\left(1-\frac{2}{\bar{C}_j}\right)+\frac{1}{\bar{C}_j}, \tag{S19}$$

where $\hat{\bar{\mu}}_{jk}$ and $\bar{C}_j$ are the function of variable ϕ_j while $\frac{C^{\mathrm{T}}_{j}\hat{\mu}^{\mathrm{T}}_{jk}-1}{C^{\mathrm{T}}_{j}-2}$ is not. The SCNA segments with the same obervation value $\hat{\bar{\mu}}_{jk}$ and $\bar{C}_j$ and the different subclonal frequency ϕ_j must obey Equation S19. We set parameter $\xi = \frac{C^{\mathrm{T}}_{j}\hat{\mu}^{\mathrm{T}}_{jk}-1}{C^{\mathrm{T}}_{j}-2}$. According to the first equation in Equation set S5 and $2 < C^{\mathrm{T}}_{j} \leqslant C_{\max}$, we have $2 < \bar{C}_j \leqslant C_{\max}$. Therefore, if $\bar{C}_j > 2$ and $\bar{C}_j \geqslant \frac{1}{\hat{\bar{\mu}}_{jk}}$, there are multiple solutions of ϕ_j in Equation set S5 on the curves of the family of function $\hat{\bar{\mu}}_{jk} = \xi\left(1-\frac{2}{\bar{C}_j}\right)+\frac{1}{\bar{C}_j}$, under the restriction of maximum absolute copy number $C_{\max}$.

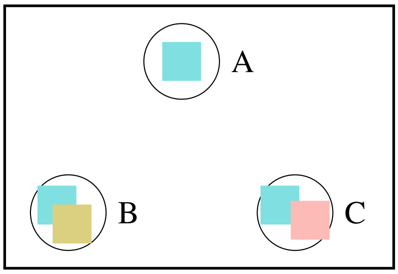

Figure S4: The relationship between subclonal population and subpopulation. In this figure, A, B and C denotes three tumor cells (the circles). In each cell, each mutation is represented as a square with a specific color. The subclonal population of the blue mutation is the set of cells that contains this blue mutation, which is $\{A, B, C\}$. The blue mutation's subclonal population contains three subpopulation. Each subpopulation contains only one cell in this figure. A mutation's subclonal population means all the cells that contains this mutation, while a subpopulation means all the cells are genetically identical in it.

4 THE VALIDATION FOR FALSE BREAKPOINTS FILTERING ALGORITHM (FBFA)

We use Pysubsim-tree[2] to simulate a tumor's next generation sequencing (NGS) read alignment data from Chromosome 1 with the evolution history configuration shown in FigureS5 and the acquired SCNA's configuration listed in Table S2. Next we simulate the true and false SCNA segments and its breakpoints by manually splitting the SCNA range in Table S2 into 1kb-length bins and randomly add 100 1kb-length normal bins to the result to account for the influence of sequencing noise and system bias on NGS based SCNA detection or segmentation tool.

The major advantage of FBFA is clustering the SCNA segments with the same NGS property to reduce the false positive SCNA segments and breakpoints. To validate the unsupervised clustering method in FBFA, we use two different parameter configuration and compare their performance with MixClone. We respectively set the total cluster number to be the maximum absolute copy number (denoted as $C_{\max}$) pre-specified and to be this value multiplies the total subclonal population number M (denoted as $M * C_{\max}$). In this section, we set $C_{\max} = 3$ and $M = 2$ according to the configuration of simulation data listed in Table S2 and Figure S5. As shown in Figure S6(a) and S7(a), SCNA segments are clustered into 3 and 6 groups respectively. The top two stripes in Figure S6(a) are clustered into one group while they are clearly separated in Figure S7(a).

FBFA selects the robust clustering algorithm 'MeanShift' [3] to cluster the B-allele frequency (BAF) value into groups in the decomposition step, because the range BAF value is fixed ($[0, 0.5]$. Although FBFA could separated part of these two stripes out as shown in Figure S6(b), there are still many SCNA segments which do not locate in the same variation clustered together. Stripe '1_0', '1_1' and '1_2' in Figure S6(b)

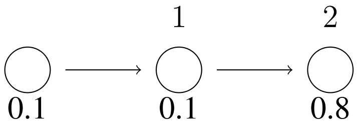

Figure S5: Evolution history configuration of simulation data. The numbers below the circles are the subpopulation frequency and the numbers above the circles are IDs for each subpopulation. The number under first circle denotes the normal contamination fraction.

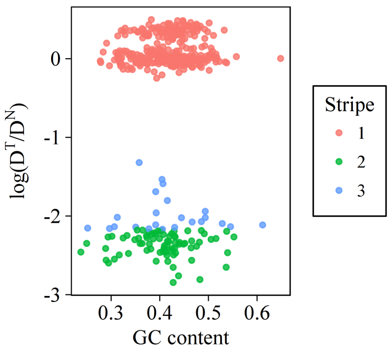

(a) Result of aggregation step of clustering of SCNA segments.

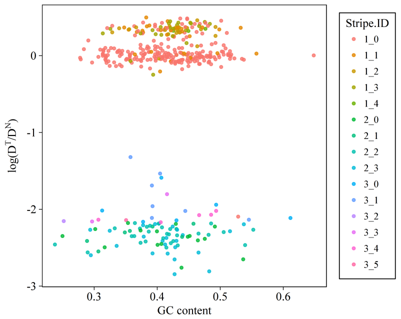

(b) Result of decomposition step of clustering of SCNA segments. In the legend, ‘Stripe.ID’ ‘1_0’∼‘1_4’ denotes the 5 sub-group of stripe ‘1’ in sub-figure(a) respectively.

Figure S6: Results of segment clustering with the total number of cluster set to be 3 in the aggregation step.

contain SCNA segments from the variation with absolute copy number 3 and the false positive SCNA (baseline segments) in each of them, while the rest stripes in Figure S6(b) contain only one type of variation in each of them. All the stripes in Figure S7(b) contains only one type of variation or contain only the false positive SCNA segments.

Figure S8 shows the result of absolute copy number obtained by FBFA and MixClone. The precision of absolute copy number estimation of FBFA with the total number of cluster set to be 3 and 6 the in aggregation step and MixClone are 0.8725, 0.98 and 0.87. The result of FBFA with the total number of cluster set to be 3 in the aggregation step is no better than the MixClone’s, although there are some SCNA segments are merged from different types of variation while clustering the segments. As shown in Figure S8(b), FBFA could more correctly estimate the absolute number if the SCNA segments with the same NGS properties are more correctly merged together.

Table S3 shows the result of subclonal population estimation of each SCNA segment. FBFA with the total number of cluster set to be 6 in the aggregation step achieves the best subclonal population estimation in comparison with the else methods. Although MixClone could accurately predict the SCNA’s subclonal population frequency with absolute copy number 0, the result of MixClone contains a lot of false prediction in baseline and deletion region.

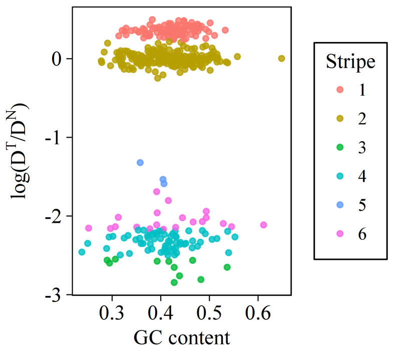

(a) Result of aggregation step of clustering of SCNA segments.

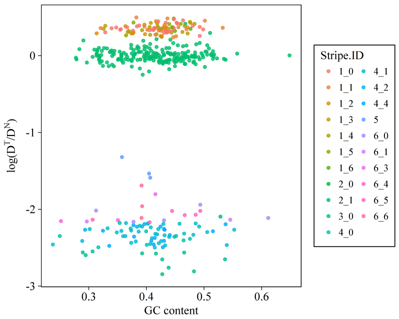

(b) Result of decomposition step of clustering of SCNA segments.In the legend, ‘Stripe.ID’ ‘1_0’∼‘1_6’ denotes the 7 sub-group of stripe ‘1’ in sub-figure(a) respectively. ‘Stripe.ID’ ‘5’ means that the stripe ‘5’ is split into only one sub-group after the decomposition step.

Figure S7: Results of segment clustering with the total number of cluster set to be 6 in the aggregation step.

We ran FBFA and MixClone on a Macbook pro Mid 2017 with Intel i5-7267U CPU and 16GB of memory. It took FBFA 97.29 and 103.04 seconds for the two parameter configurations while it took Mixclone 6202.73 seconds on the same computer.

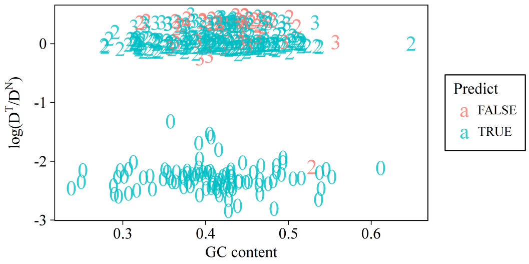

(a) false breakpoints filtering algorithm (FBFA) with the total number of cluster set to be 3 in the aggregation step

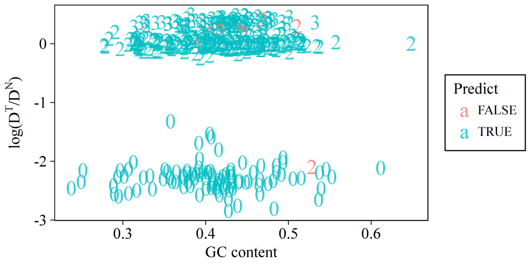

(b) FBFA with the total number of cluster set to be 6 in the aggregation step

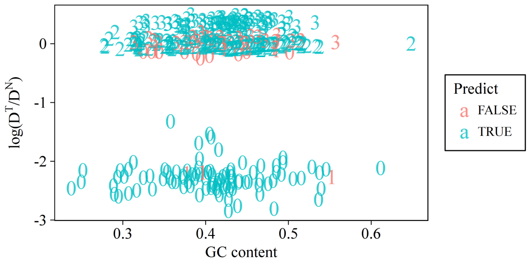

(c) MixClone

Figure S8: Result of absolute copy number estimation

Table S1. The $\bar{C}_j$ and $\hat{\bar{\mu}}_{jk}$ ranges for unique solution and multiple solutions given $C_j^{\mathrm{T}} \leqslant 15$

ξ	One solution		Multiple solutions	
	$\bar{C}_j$	$\hat{\bar{\mu}}_{jk}$	$\bar{C}_j$	$\hat{\bar{\mu}}_{jk}$
0.0000	$[15, 14)$	$[0.0667, 0.0714)$	$[14, 2)$	$[0.0714, 0.5000)$
-0.5000	$[4, 2)$	$[0.0000, 0.5000)$	–	–
0.1538	$[15, 2)$	$[0.2000, 0.5000)$	–	–
-0.3333	$[5, 2)$	$[0.0000, 0.5000)$	–	–
-0.1250	$[10, 2)$	$[0.0000, 0.5000)$	–	–
0.4615	$[15, 2)$	$[0.4667, 0.5000)$	–	–
-0.1111	$[11, 2)$	$[0.0000, 0.5000)$	–	–
0.1818	$[13, 2)$	$[0.2308, 0.5000)$	–	–
-0.0833	$[14, 2)$	$[0.0000, 0.5000)$	–	–
-0.2000	$[7, 2)$	$[0.0000, 0.5000)$	–	–
-0.1000	$[12, 2)$	$[0.0000, 0.5000)$	–	–
0.5000	$[14, 12)$	$[0.5000, 0.5000)$	$[12, 2)$	$[0.5000, 0.5000)$
0.3077	$[15, 2)$	$[0.3333, 0.5000)$	–	–
0.2222	$[11, 2)$	$[0.2727, 0.5000)$	–	–
0.4167	$[14, 2)$	$[0.4286, 0.5000)$	–	–
-0.1667	$[8, 2)$	$[0.0000, 0.5000)$	–	–
-0.0909	$[13, 2)$	$[0.0000, 0.5000)$	–	–
-0.0769	$[15, 2)$	$[0.0000, 0.5000)$	–	–
0.2857	$[9, 2)$	$[0.3333, 0.5000)$	–	–
0.4444	$[11, 2)$	$[0.4545, 0.5000)$	–	–
0.3636	$[13, 2)$	$[0.3846, 0.5000)$	–	–
0.4545	$[13, 2)$	$[0.4615, 0.5000)$	–	–
0.1429	$[9, 2)$	$[0.2222, 0.5000)$	–	–
-0.1429	$[9, 2)$	$[0.0000, 0.5000)$	–	–
0.3000	$[12, 2)$	$[0.3333, 0.5000)$	–	–
0.1000	$[12, 2)$	$[0.1667, 0.5000)$	–	–
0.0769	$[15, 2)$	$[0.1333, 0.5000)$	–	–
0.0909	$[13, 2)$	$[0.1538, 0.5000)$	–	–
0.3333	$[14, 11)$	$[0.3571, 0.3636)$	$[11, 2)$	$[0.3636, 0.5000)$
0.2727	$[13, 2)$	$[0.3077, 0.5000)$	–	–
0.1250	$[10, 2)$	$[0.2000, 0.5000)$	–	–
0.3846	$[15, 2)$	$[0.4000, 0.5000)$	–	–
0.4000	$[12, 7)$	$[0.4167, 0.4286)$	$[7, 2)$	$[0.4286, 0.5000)$
0.2000	$[12, 7)$	$[0.2500, 0.2857)$	$[7, 2)$	$[0.2857, 0.5000)$
-0.2500	$[6, 2)$	$[0.0000, 0.5000)$	–	–
0.2308	$[15, 2)$	$[0.2667, 0.5000)$	–	–
0.4286	$[9, 2)$	$[0.4444, 0.5000)$	–	–
0.0833	$[14, 2)$	$[0.1429, 0.5000)$	–	–
0.1111	$[11, 2)$	$[0.1818, 0.5000)$	–	–
0.1667	$[14, 8)$	$[0.2143, 0.2500)$	$[8, 2)$	$[0.2500, 0.5000)$
0.2500	$[14, 10)$	$[0.2857, 0.3000)$	$[10, 2)$	$[0.3000, 0.5000)$
-1.0000	$[3, 2)$	$[0.0000, 0.5000)$	–	–
0.3750	$[10, 2)$	$[0.4000, 0.5000)$	–	–

Table S2. The acquired SCNA's configuration for each subpopulation of the simulation data.

ID	chrome	position	copy number	genotype	subclone frequency
1	chr1	210682863	3	PPM	0.8
2	chr1	152728665	0	$\emptyset$	0.9

Table S3. Result of subclonal population frequency estimation. 'FBFA_3' and 'FBFA_6' denote the FBFA with the total number of cluster set to be 3 and 6 in the aggregation step respectively.

	100 segments in #1 SCNA ($\phi = 0.8, C = 3$)	100 segments in #2 SCNA ($\phi = 0.9, C = 0$)	200 baseline segments ($\phi = 1, C = 2$)
FBFA_3	$\frac{99}{(\phi=0.877)} + \frac{1}{(\phi=1)}$	$\frac{39}{(\phi=0.877)} + \frac{26}{(\phi=0.99)} + \frac{35}{(\phi=1)}$	$\frac{13}{(\phi=0.877)} + \frac{2}{(\phi=0.99)} + \frac{185}{(\phi=1)}$
FBFA_6	$\frac{75}{(\phi=0.857)} + \frac{19}{(\phi=0.9)} + \frac{6}{(\phi=1)}$	$\frac{22}{(\phi=0.857)} + \frac{77}{(\phi=0.9)} + \frac{1}{(\phi=1)}$	$\frac{1}{(\phi=0.857)} + \frac{199}{(\phi=1)}$
MixClone	$\frac{100}{(\phi=0.989)}$	$\frac{98}{(\phi=0.989)} + \frac{2}{(\phi=0.148)}$	$\frac{72}{(\phi=0.148)} + \frac{1}{(\phi=0.898)} + \frac{127}{(\phi=1)}$

REFERENCES

[1]Yuval Benjamini and Terence P Speed. Summarizing and correcting the gc content bias in high-throughput sequencing. *Nucleic acids research*, 40(10):e72–e72, 2012.

[2]Yanshuo Chu, Ling Wang, Rongjie Wang, Mingxiang Teng, and Yadong Wang. Pysubsim-tree: A package for simulating tumor genomes according to tumor evolution history. In *Bioinformatics and Biomedicine (BIBM), 2017 IEEE International Conference on*, pages 2195–2197. IEEE, 2017.

[3]Dorin Comaniciu and Peter Meer. Mean shift: A robust approach toward feature space analysis. *IEEE Transactions on pattern analysis and machine intelligence*, 24(5):603–619, 2002.

[4]Arief Gusnanto, Henry M Wood, Yudi Pawitan, Pamela Rabbitts, and Stefano Berri. Correcting for cancer genome size and tumour cell content enables better estimation of copy number alterations from next-generation sequence data. *Bioinformatics*, 28(1):40–47, 2012.

[5]Yi Li and Xiaohui Xie. Mixclone: a mixture model for inferring tumor subclonal populations. *BMC genomics*, 16(Suppl 2):S1, 2015.

[6]M Elizabeth O Locke, Maja Milojevic, Susan T Eitutis, Nisha Patel, Andrea E Wishart, Mark Daley, and Kathleen A Hill. Genomic copy number variation in mus musculus. *BMC genomics*, 16(1):497, 2015.

[7]Layla Oesper, Ahmad Mahmoody, and Benjamin J Raphael. Theta: inferring intra-tumor heterogeneity from high-throughput dna sequencing data. *Genome biology*, 14(7):1, 2013.

[8]Ryan Prescott Adams, Zoubin Ghahramani, and Michael I Jordan. Tree-structured stick breaking processes for hierarchical data. *arXiv preprint arXiv:1006.1062*, 2010.

[9]Ruibin Xi, Joe Luquette, Angela Hadjipanayis, Tae-Min Kim, and Peter J Park. Bic-seq: a fast algorithm for detection of copy number alterations based on high-throughput sequencing data. *Genome biology*, 11(1):1, 2010.
